# Gene activation via Cre/*lox*-mediated excision in cowpea (*Vigna unguiculata*)

**DOI:** 10.1007/s00299-021-02789-z

**Published:** 2021-09-30

**Authors:** Zhifen Zhang, Yinping Guo, Kathleen Monfero Marasigan, Joann A. Conner, Peggy Ozias-Akins

**Affiliations:** Department of Horticulture and Institute of Plant Breeding, Genetics and Genomics, University of Georgia, 2356 Rainwater Rd, Tifton, GA 31793 USA

**Keywords:** Cre/*lox*, Promoter, *AtRps5a*, *AtDD45*, Cowpea, Tobacco

## Abstract

**Key message:**

Expression of Cre recombinase by *AtRps5a*_*pro*_ or *AtDD45*_*pro*_ enabled Cre/*lox*-mediated recombination at an early embryonic developmental stage upon crossing, activating transgenes in the hybrid cowpea and tobacco.

**Abstract:**

Genetic engineering ideally results in precise spatiotemporal control of transgene expression. To activate transgenes exclusively in a hybrid upon fertilization, we evaluated a Cre/*lox*-mediated gene activation system with the *Cre* recombinase expressed by either *AtRps5a* or *AtDD45* promoters that showed activity in egg cells and young embryos. In crosses between *Cre* recombinase lines and transgenic lines harboring a *lox*-excision reporter cassette with *ZsGreen* driven by the *AtUbq3* promoter after Cre/*lox*-mediated recombination, we observed complete excision of the *lox*-flanked intervening DNA sequence between the *AtUbq3*_*pro*_ and the *ZsGreen* coding sequence in F_1_ progeny upon genotyping but no *ZsGreen* expression in F_1_ seeds or seedlings. The incapability to observe ZsGreen fluorescence was attributed to the activity of the *AtUbq3*_*pro*_. Strong *ZsGreen* expression in F_1_ seeds was observed after recombination when *ZsGreen* was driven by the *AtUbq10* promoter. Using the *AtDD45*_*pro*_ to express *Cre* resulted in more variation in recombination frequencies between transgenic lines and crosses. Regardless of the promoter used to regulate *Cre*, mosaic F_1_ progeny were rare, suggesting gene activation at an early embryo-developmental stage. Observation of *ZsGreen*-expressing tobacco embryos at the globular stage from crosses with the *AtRps5a*_*pro*_*Cre* lines pollinated by the *AtUbq3*_*pro*_*lox* line supported the early activation mode.

**Supplementary Information:**

The online version contains supplementary material available at 10.1007/s00299-021-02789-z.

## Introduction

Precise gene regulation is critical in genetic engineering. Conditional transgene activation usually is preferred since ectopic expression of transgenes may result in undesired phenotypes. The application of tissue-specific promoters or inducible promoters is a common approach to achieve conditional transgene activation. Using two-component systems for gene activation wherein the transgene remains inactive until both the components converge in the same cell can offer an alternative switch perspective. With the “activator” regulated by a tissue-specific or inducible promoter, a two-component system potentially can lead to tighter control of spatiotemporal transgene expression.

The Cre/*lox* system is one of the most well-characterized two-component systems and has been broadly used in eukaryotic organisms, including plants (Branda and Dymecki 2004; Gilbertson 2003; Srivastava and Thomson 2016). In the Cre/*lox* system, a 38-kDa recombinase Cre from bacteriophage P1 can recognize a 34-bp *lox* (locus of crossover) site by binding to the inverted repeats and effecting recombination at the 8-bp spacer inside the *lox* site (Van Duyne 2001). Depending on the numbers of the *lox* sites employed and their orientations, Cre/*lox*-mediated recombination can result in excision, inversion, integration, or sequence exchange between two nonhomologous chromosomes (Branda and Dymecki 2004; Srivastava and Thomson 2016). The excision reaction has been the basis of gene activation (Odell et al. 1994; Zhang et al. 2003; Heidstra et al. 2004) and inactivation (Russell et al. 1992; Kopertekh et al. 2010; Lowe et al. 2016) through Cre/*lox*-mediated recombination. For gene activation, an intervening DNA sequence flanked by two *lox* sites in the same orientation is placed between the promoter and coding sequence (CDS) of the gene of interest (GOI) so that the GOI remains inactive until the Cre recombinase excises the *lox*-flanked intervening sequence. The recombination results in a circular DNA molecule of the intervening sequence and leaves one *lox* site as a “scar” between the promoter and CDS. Gene activation by Cre/*lox*-mediated recombination has been evaluated in plants with the *Cre* gene regulated by a constitutive promoter (Bayley et al. 1992; Hoa et al. 2002; Zhang et al. 2003). Conditional gene activation in *lox*-containing plants also was achieved using inducible promoters (Hoff et al. 2001; Zuo et al. 2001) or tissue-specific promoters (Odell et al. 1994; Chen et al. 2017) to express *Cre*. To activate the GOI in crosses between *Cre*- and *lox*-containing lines, promoters active in reproductive tissues are preferable for *Cre* expression, as the presence of Cre recombinase in gametes presumably enables excision of the *lox*-flanked intervening sequence upon fertilization and activates the GOI as early as possible. Delayed expression of the *Cre* gene likely results in mosaic embryos as observed previously when the *Cre* gene was regulated by seed-specific promoters that became active at the heart stage of embryo development (Odell et al. 1994).

Careful selection of promoters that express *Cre* in gametes or zygotes presumably will be able to activate the GOI at the zygote stage or at least an early embryo-developmental stage. Several reproduction-specific promoters, such as flower (Bai et al. 2008; Verweire et al. 2007), pollen (Luo et al. 2007; Mlynárová et al. 2006; Verweire et al. 2007), and pollen-and-embryo promoters (Polóniová et al. 2015) have been evaluated to express *Cre* for marker-gene excision but never for gene activation. Moreover, the promoters tested were predominantly male gamete-specific, probably because marker-gene excision generally intends to prevent unwanted gene flow to non-transgenic plants via pollen and seeds. The use of female gamete-specific promoters to express *Cre* remains unreported. The promoter of *Arabidopsis RIBOSOMAL PROTEIN S5A* (*AtRps5a*) can bring about a visible expression of marker genes in the egg cells and sperm cell nuclei before fertilization (Maruyama et al. 2013) and strong expression was found in young embryos from the zygote stage up until the globular stage (Weijers et al. 2001; Maruyama et al. 2013; Gooh et al. 2015). The activity in egg cells and young embryos makes the *AtRps5a*_*pro*_ a candidate for expressing *Cre* to activate a GOI upon fertilization. However, the *AtRps5a*_*pro*_ also showed high activity in the floral meristem, shoot/root apical meristem, and leaf primordia (Weijers et al. 2001). It is unknown whether continuous high expression of the DNA-binding protein Cre in these tissues could cause abnormal phenotypes as suggested in some species (Coppoolse et al. 2003). As a precaution, additional tissue-specific promoters, such as the promoter of *Arabidopsis DOWN REGULATED IN DETERMINANT INFERTILE (DD) 45/EGG CELL-SECRETED (EC) 1.2* (*AtDD45/EC1.2*), can be tested for providing an alternative to the *AtRps5a*_*pro*_. The *AtDD45/EC1.2*_*pro*_ (referred to as *AtDD45*_*pro*_ for short in the remaining text) can drive gene expression specifically in egg cells before fertilization (Steffen et al. 2007; Sprunck et al. 2012) and express the transgene in early developing embryos no later than the eight-cell stage after fertilization (Lawit et al. 2013). It is unknown yet whether shortening the expression time of *Cre* with the *AtDD45*_*pro*_ will lead to lower recombination efficiency and more mosaic F_1_ embryos than with the *AtRps5a*_*pro*_.

Cowpea (*Vigna unguiculata* [L.] Walp.) is one of several important crop plants for food security in sub-Saharan Africa, given its resilience to drought and high temperature (Singh 2014; Carvalho et al. 2017). As a legume, cowpea can also tolerate low-fertility soil thanks to its capability to establish a symbiotic association with nitrogen-fixing bacteria and vesicular-arbuscular mycorrhizal fungi. Cowpea also is an ideal niche crop for cereal-based multiple cropping systems considering the availability of early-maturing and pest-resistant varieties (Singh 2014), and its importance may increase around the globe under changing climate conditions. Currently, cowpea is mostly grown in tropical and subtropical regions and consumed in the human diet or used as fodder in West and Central Africa, Europe, Asia, and America. The increase in cowpea production and consumption has been considerable (Singh 2014; FAOSTAT 2018) and improvement in cowpea varieties has been made in the last several decades (Boukar et al. 2020; Singh 2014). Cowpea improvement likely will accelerate thanks to the availability of new genomic and transcriptomic resources (Huynh et al. 2018; Lonardi et al. 2019; Muñoz-Amatriaín et al. 2017; Spriggs et al. 2018; Yao et al. 2016) as well as an efficient transformation method (Che et al. 2021) that enables transgene introduction and genome editing. The novelty of the current study is in demonstrating the efficacy of the Cre/*lox* system for gene activation in early embryo development through the use of early embryo and female gametophyte promoters *AtRps5a*_*pro*_ and *AtDD45*_*pro*_. The results will pave the way for utilizing the Cre/lox system to activate transgenes conditionally in cowpea.

## Materials and methods

### *Cre* and *lox*-reporter gene constructs

**Fig. 1:**
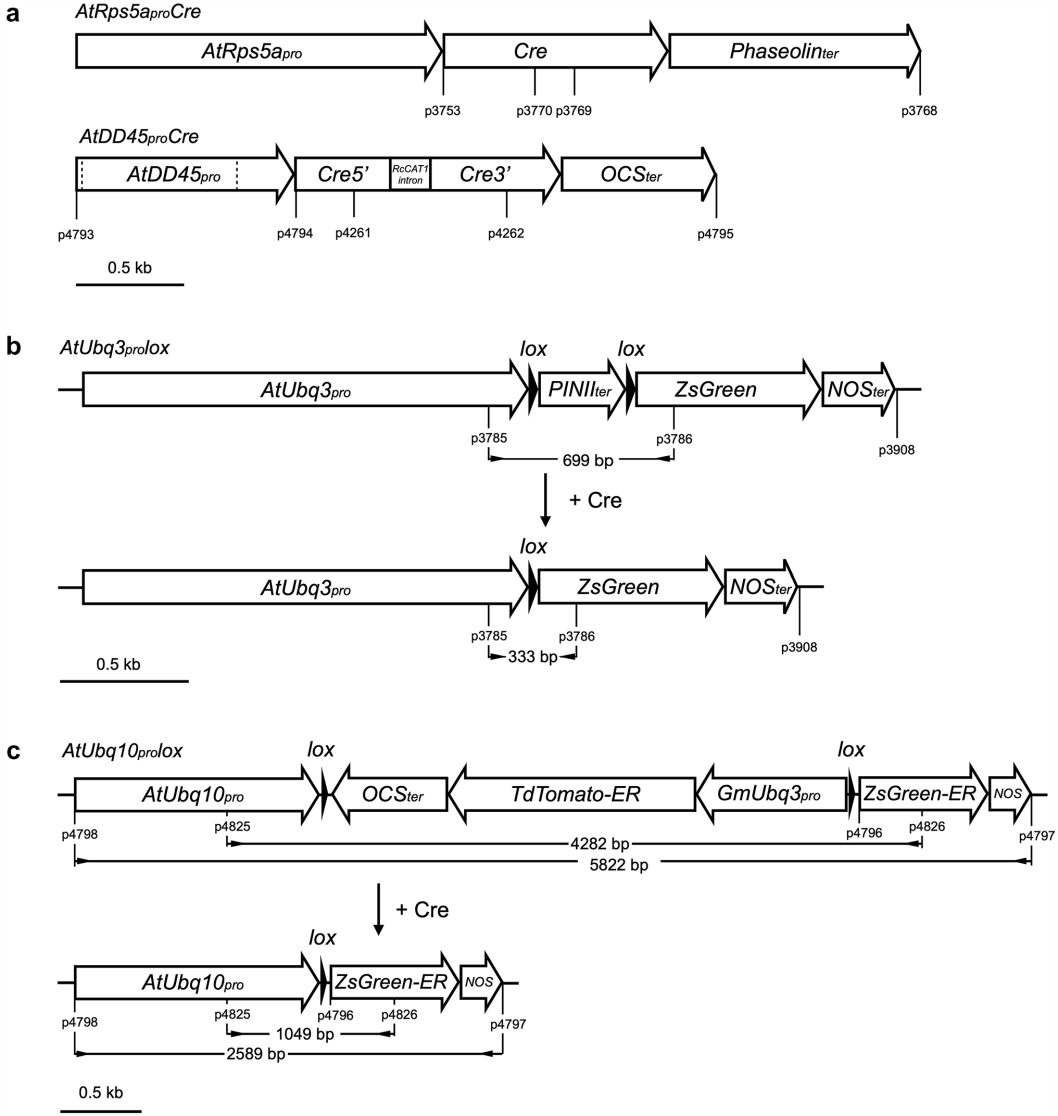
Four cassettes used to evaluate Cre/*lox*-mediated recombination. **a** Cassettes *AtRps5a*_*pro*_*Cre* and *AtDD45*_*pro*_*Cre*, dash lines in the *AtDD45*_*pro*_ indicate a “T” to “A” mutation and a “G” to “T” mutation created respectively at −264 bp and −979 bp upstream from the start site of *Cre* during synthesis to facilitate the cassette assembly using Golden Gate Cloning (Engler et al. 2008); **b**, **c** diagrams of two inactive *lox*-reporter cassettes and their activated versions after Cre/*lox*-mediated recombination; **b** cassette *AtUbq3*_*pro*_*lox*, the 8-bp spacer in the *lox* sites was oriented as its reverse complement counterpart (Albert et al. 1995) so that the two ATGs in the spacer were in the reverse orientation with respect to the promoter; **c** cassette *AtUbq10*_*pro*_*lox*, *ZsGreen-ER* and *tdTomato-ER* are *ZsGreen* and *tdTomato* containing an endoplasmic reticulum signal peptide (ERSP) at the 5′ end and a KDEL-ER retention motif at the 3′ end, respectively; the sizes shown are the fragment amplified by PCR with the specific primers (see Table S3).

To test the efficacy of gene activation via Cre/*lox*-mediated excision in tobacco and cowpea, we first constructed two cassettes *AtRps5a*_*pro*_*:Cre:phaseolin*_*term*_ (*AtRps5a*_*pro*_*Cre*), and *AtUbq3*_*pro*_*:lox-PINII*_*term*_*-lox:ZsGreen:NOS*_*term*_ (*AtUbq3*_*pro*_*lox*). In the cassette *AtRps5a*_*pro*_*Cre* (Fig. 1a), the *Cre* gene was controlled by the promoter (1684 bp) from *Arabidopsis thaliana ribosomal protein subunit 5a* (*AtRps5a*, AT3G11940; Weijers et al. 2001) and the *phaseolin* 3’ terminator. In the cassette *AtUbq3*_*pro*_*lox* (Fig. 1b), the *ZsGreen* gene was controlled by the promoter (1721 bp) from *A. thaliana polyubiquitin 3* (*AtUbq3*, AT5G03240, including its 5′ UTR and leading intron) and the *nopaline synthase* (*NOS*) terminator. A potato Proteinase Inhibitor II (*PINII*) terminator (Keil et al. 1986) flanked by two modified *lox* sites was inserted between the *AtUbq3*_*pro*_ and *ZsGreen* coding sequence (CDS). The cassette *AtRps5a*_*pro*_*Cre*, together with a fluorescent-marker cassette *GmEF1a*_*pro*_*:DsRed:NOS*_*term*_ (*GmEF1a*_*pro*_*DsRed*) and a selectable-marker cassette, was transferred into the T-DNA region of a binary vector pPZP201BK (Covert et al. 2001) (Table S1). In the cassette *GmEF1a*_*pro*_*DsRed*, a *DsRed* gene was controlled by the promoter (2166 bp) from soybean (*Glycine max*) *elongation factor 1a* (*GmEF1a*, Glyma.17G186600, including its 5′ UTR and leading intron; Li et al. 2015) and the *NOS* terminator. In the selectable-marker cassette, the selectable marker gene was controlled by the promoter (930 bp) from potato (*Solanum tuberosum*) *polyubiquitin* (*StUbq*, GenBank: L22576; Garbarino and Belknap 1994) and the *NOS* terminator. Flanked by the same promoter and terminator, three selectable-marker CDS, *neomycin phosphotransferase II* (*nptII*), *hygromycin phosphotransferase* (*hpt*), and *Bialaphos resistance* (*bar*), were evaluated side by side for their effect on the efficiency of cowpea transformation. The cassette *AtUbq3*_*pro*_*lox* was transferred into the T-DNA region of the binary vector pORE (Coutu et al. 2007), together with a kanamycin resistance cassette (*S1*_*pro*_*nptII*) wherein the *nptII* gene containing a *Ricinus communis catalase 1* (*CAT1*) intron was controlled by the *S1* promoter and *S3* terminator from subterranean clover stunt virus (SCSV) (Schünmann et al. 2003) (Table S1). The cassettes *AtUbq3*_*pro*_*lox* and *S1*_*pro*_*nptII* were also transferred into the T-DNA region of the binary vector pPZP201BK, together with the cassette *GmEF1a*_*pro*_*DsRed* as a fluorescent marker (Table S1).

To further evaluate Cre/*lox*-mediated gene activation in cowpea, two additional cassettes were constructed as *AtDD45*_*pro*_*:Cre:OCS*_*term*_ (*AtDD45*_*pro*_*Cre*) and *AtUbq10*_*pro*_*:loxP-GmUbq3*_*pro*_*:tdTomatoER:OCS-loxP:ZsGreen:NOS*_*term*_ (*AtUbq10*_*pro*_*lox*). In the cassette *AtDD45*_*pro*_*Cre* (Fig. 1a), the synthetic codon-optimized *Cre* gene containing a modified *CAT1* intron was controlled by the promoter (1002 bp) from *A. thaliana DOWN REGULATED IN DETERMINANT INFERTILE* (*DD*) 45/*Egg Cell-secreted protein 1.2* (*AtDD45/EC1.2*, AT2G21740; Steffen et al. 2007; Sprunck et al. 2012) and the *octopine synthase* (*OCS*) terminator. In the cassette *AtUbq10*_*pro*_*lox* (Fig. 1c), the synthetic codon-optimized *ZsGreen* gene was controlled by the promoter (1500 bp) from *A. thaliana polyubiquitin 10* (*AtUbq10*, AT4G05320, including its 5’UTR and leading intron) and the *NOS* terminator. Between the *AtUbq10*_*pro*_ and the *ZsGreen* CDS, a cassette *GmUbq3*_*pro*_*:tdTomatoER:OCS*_*term*_ (*GmUbq3*_*pro*_*tdTomato*) flanked by two *loxP* (the original *lox* from bacteriophage P1) sites was inserted in a reverse direction for the *AtUbq10* promoter. The *tdTomato* gene (a variant of *DsRed*) (Shaner et al. 2004) was controlled by the promoter (919 bp) from soybean (*G. max*) *ubiquitin 3* (*GmUbq3*, Glyma.20G141600, including its 5’UTR and leading intron) and the *OCS* terminator (Fig. 1c). The cassettes *AtDD45*_*pro*_*Cre* and *AtUbq10*_*pro*_*lox* were transferred respectively into the T-DNA region of the binary vector pAGM4673 (Weber et al. 2011), together with a spectinomycin resistance cassette (Che et al. 2021) (Table S1). Considering that the *35S* enhancer in the spectinomycin resistance cassette might interact with the *AtDD45*_*pro*_ in the cassette *AtDD45*_*pro*_*Cre* and affect its tissue-specificity, a 2 kb transformation booster sequence (TBS) (Singer et al. 2011) was placed between the cassette *AtDD45*_*pro*_*Cre* and the spectinomycin resistance cassette (Table S1).

### Transient assay by microprojectile bombardment

Mature cowpea seeds were surface sterilized with 10% commercial bleach (6% (w/v) sodium hypochlorite; Clorox, Oakland, CA, USA) for 30 min with agitation at 150 rpm, followed by rinsing with sterilized reverse osmosis deionized (RODI) water at least five times. After sterilization, seeds were immersed in sterilized RODI water overnight. Cotyledons were excised from the imbibed seeds and the embryo axes were used for microprojectile bombardment. With one side touching the medium, ten embryo axes were laid within a circle of 2 cm in diameter at the center of a Petri dish containing 0 MS medium composed of 1× Murashige and Skoog (MS) salts and vitamins, 3% (w/v) sucrose (Research Products International, Mt Prospect, IL, USA), and 8 mg/l agarose (pH 5.7). Microprojectile bombardment was conducted using a PDS-1000/He^TM^ system (Bio-Rad Laboratories, Hercules, CA, USA) under 1550 psi helium in a vacuum of 23 in Hg, with the Petri dish carrying embryo axes positioned on the sample platform 5 cm below the launch assembly. Each bombardment delivered approximately 156 ng DNA composed of an equal molar amount of the *Cre* cassette and *lox*-reporter gene cassette and 50 µg gold microcarriers 0.6 µm in diameter (Bio-Rad Laboratories). Each sample was bombarded twice. Upon evaluating the cassettes *AtRps5a*_*pro*_*Cre* and *AtUbq3*_*pro*_*lox* harbored in the plasmid pBlueScript KS(+), a trace amount of the cassette *GmEF1a*_*pro*_*DsRed* harbored in the plasmid pBlueScript KS(+) also was added so that we could check the quality of each bombardment. With the *lox*-flanked *GmUbq3*_*pro*_*tdTomato* in the *lox*-reporter cassette, the cassette *GmEF1a*_*pro*_*DsRed* was omitted upon evaluating the cassette *AtDD45*_*pro*_*Cre* in vector RC2677 and the cassette *AtUbq10*_*pro*_*lox* in vector RC2717 (Table S1). Cowpea embryo axes were observed for *ZsGreen* expression 24 h after bombardment.

### *Agrobacterium* inoculum and plant transformation

All recombinant binary vectors (Table S1) were introduced into competent cells of *Agrobacterium* strain AGL1 using the freeze-thaw method (Chen et al. 1994). *Agrobacterium* carrying the recombinant vectors was stored in 15% glycerol at −80 °C. For tobacco transformation and cowpea transformation using Method 1 and 2 (see below), *Agrobacterium* inoculum was prepared as follows: *Agrobacterium* from the glycerol stocks was cultured in 2-ml liquid Lysogeny broth (LB) medium (ThermoFisher Scientific, Waltham, MA, USA) containing 100 mg/l kanamycin, 50 mg/l rifampicin, and 50 mg/l carbenicillin, with incubation at 150 rpm and 28 °C for 24 h. The bacterial culture was diluted 1:100 with liquid LB medium containing the same antibiotics and incubated overnight at 150 rpm, 28 °C. The culture was centrifuged for 10 min at 6000 rpm using a J2-21M centrifuge (Beckman Coulter, Brea, CA, USA), and re-suspended in liquid co-culture medium, with OD_600_ adjusted to 0.5–0.6. For cowpea transformation using Method 3, *Agrobacterium* inoculum was prepared according to Che et al. (2021).

The *AtRps5a*_*pro*_*Cre* and *AtUbq3*_*pro*_*lox* were introduced into tobacco PI 552484 (seeds purchased from Lehle Seeds, Round Rock, TX, USA) following the protocol of Clemente (2006) with some modifications (Zhang et al. 2020). Transgenic plants containing either the cassette *AtRps5a*_*pro*_*Cre* or *AtUbq3*_*pro*_*lox* were recovered with selection on either 20 mg/l hygromycin or 200 mg/l kanamycin, according to the selectable marker in the plasmids used for transformation (Table S1). Plants derived from different leaf disc explants were independent, while those from the same explants were potentially the same lines.

Different *Cre* or *lox*-reporter cassettes were introduced into cowpea IT86D-1010 following three distinct methods. The cassettes *AtRps5a*_*pro*_*Cre* and *AtUbq3*_*pro*_*lox* were introduced by either Method 1 described by Popelka et al. (2006) with some modification or Method 2 wherein explants were cotyledonary nodes from seedlings pre-conditioned with 5 mg/l 6-benzylaminopurine (BA). Both methods started with mature cowpea seeds surface sterilized as previously described (Sect. “Transient assay by microprojectile bombardment”). In Method 1, sterilized seeds were immersed in sterilized RODI water overnight. The cotyledonary nodes from the imbibed seeds were used as explants for *Agrobacterium*-mediated transformation after removing the seed coat, shoot tips, cotyledons, and radicals. Every ten explants were immersed in 2-ml inoculum consisting of *Agrobacterium* suspended in a liquid co-culture medium (CCM, Table S2) supplemented with 0.02% (v/v) Silwet-77 (Lehle Seeds, Round Rock, TX, USA) in a 10-ml borosilicate glass test tube (Cat# 14-961-27, ThermoFisher Scientific, Waltham, MA, USA), then sonicated for 20 s using an FS30H sonicator (ThermoFisher Scientific). After 30 min incubation at room temperature, explants were blotted dry with sterile filter paper and transferred onto CCM (Table S2) with a piece of filter paper on top of the medium to prevent direct contact between explants and the medium, 20–25 explants each plate. In Method 2, sterilized mature seeds were cultured on a germination medium (GM, Table S2) for 4 d. Seedlings with a greening cotyledonary node were selected, followed by excision of cotyledons, shoot tips, and roots. The remaining cotyledonary nodes were used as explants for transformation. Every five explants were immersed in 2-ml inoculum consisting of *Agrobacterium* resuspended in a liquid modified co-culture medium (CCM’, Table S2) supplemented with 0.02% (v/v) Silwet-77 in a 10-ml borosilicate glass test tube then sonicated for 4 min. After 30-min incubation at room temperature, explants were blotted dry and placed on CCM’ plates (Table S2), 12 explants each, with a piece of filter paper between explants and the medium. In both methods, after a 5-day co-culture, explants were washed in a liquid shoot induction medium (SIM, Table S2) then blotted dry. Explants were transferred to SIM (Table S2) supplemented with 20 mg/l hygromycin, 5 mg/l phosphinothricin (PPT), or 200 mg/l kanamycin according to the selectable marker gene used in the vectors (Table S1). Explants forming shoots were transferred to fresh SIM every other week and monitored for *DsRed* expression. Explants with transgenic shoots were transferred to a shoot elongation medium (SEM, Table S2) supplemented with the same selective agent used in SIM for shoot development and rooting.

Transgenic shoots that failed to form roots were recovered by in vitro grafting adapted from a method for sunflower (Zhang and Finer 2016). Sterilized mature seeds were germinated under a sterilized folded paper towel saturated with sterile RODI water in Magenta^TM^ GA7 vessels (Sigma-Aldrich, St Louis, MO, USA), with ten seeds in each box. Rootstocks were made from 5-day-old seedlings by removing the shoot tips and cotyledons. As a scion, a developed transgenic shoot (> 0.5 cm) with its base shaped into a wedge was inserted into the longitudinal incision (0.5–1 cm) made in the side of the rootstock’s hypocotyl. The hypocotyl tissue from both sides held the scion in place. The grafted plants were grown in GA7 vessels containing 0MS medium supplemented with 30 mg/l meropenem (ABBLIS Chemicals, Houston, TX, USA) for 2–3 weeks. Elongation of the scions indicated graft success. The successfully rooted or grafted plants were transferred to soil, acclimatized in plastic containers with the lids gradually opened over a 3-week period. Plants were transferred to the greenhouse to set seeds when they were sufficiently hardened and growing vigorously.

The cassettes *AtDD45*_*pro*_*Cre* and *AtUbq10*_*pro*_*lox* were introduced into cowpea using Method 3 (Che et al. 2021) given its higher efficiency in recovering transgenic plants. In brief, mature seeds were surface-sterilized overnight using chlorine gas, then soaked overnight in a bean germination medium (Che et al. 2021). Embryo axes were isolated by removing seed coats, cotyledons, and plumules without damaging the meristematic dome and collected in sterile RODI water. After removing water, embryo axes (100–200 pieces) were infected with 15-ml *Agrobacterium* inoculum plus 50-μl sterile Poloxamer 188 10% solution in a 100 × 25 mm Petri dish (ThermoFisher Scientific). After sonication for 3 s, 10 ml of inoculum was added to each Petri dish and incubated at 60 rpm, room temperature, for 1.5 h in the dark. Explants were removed from inoculum and transferred to filter paper (VWR Cat# 28320-020) wetted with 700 μl of infection medium (Che et al. 2021) in a 100 × 25 mm Petri dish, with every 30 explants in a pile. After 2-day co-culture at 21 °C under dim light with a 16/8 h light cycle, embryo axes were inserted into SIM (Table S2) supplemented with 50 mg/l spectinomycin with shoot apex and cotyledonary node above the medium. The shoot apex was removed after 5-day incubation on SIM to facilitate the formation of axillary shoots. After 4 weeks, transgenic shoots were either transferred to a rooting medium (Che et al. 2021) for rooting or a shoot elongation medium (Che et al. 2021) for further growth before transfer to the rooting medium. Plantlets derived from different cotyledonary-node explants were independent, while those from the same explants were considered as potentially the same lines.

All plant tissues were incubated at 25 °C, with a 16/8 h light cycle, except for the cowpea transformation using Method 3 wherein plant tissues were incubated at 25 °C and 24-h light after co-culture. MS salts, MS vitamins, and acetosyringone (AS) were from PhytoTechnology Laboratories (Lenexa, KS, USA). Unless otherwise noted, all chemicals were from Millipore Sigma (St. Louis, MO, USA).

### Genotyping

Genomic DNA of tobacco and cowpea was extracted from leaf tissue using the cetyltrimethylammonium bromide (CTAB) method (Doyle and Doyle 1990). The presence of the transgene was verified by polymerase chain reaction (PCR) using corresponding primer combinations (Table S3). We conducted PCR using PrimeStar GXL DNA polymerase (Takara Bio, Kusatsu, Japan) in a 25 µl reaction for amplicons larger than 1 kb and using GoTaq^®^ Master Mix (M7123, Promega, Madison, WI, USA) in a 20 µl reaction for amplicons smaller than 1 kb. The PCR setup followed the manufacturers' instructions with 20–100 ng genomic DNA as a template. PCR products were visualized under UV light after electrophoresis in 1% agarose gels and staining with ethidium bromide.

### Evaluation of transgene segregation in transgenic lines

In tobacco, after surface sterilization according to Zhang et al. (2020), T1 seeds of the lines harboring the cassette *AtRps5a*_*pro*_*Cre* were germinated on 0MS medium containing either 200 mg/l kanamycin or 20 mg/l hygromycin depending on the selectable marker used in the vectors (Table S1). After 2 weeks, seedlings were observed for *DsRed* expression and counted. Surface-sterilized T1 seeds of tobacco lines harboring the cassette *AtUbq3*_*pro*_*lox* (from vector pZZ017, Table S1) were germinated on 0MS medium containing 600 mg/l kanamycin for a stricter selection to avoid possible false positives. After 2 weeks, the green seedlings were counted as transgenic while the seedlings showing chlorosis at cotyledons or the shoot apex were counted as non-transgenic.

In cowpea, mature T1 seeds of the lines harboring the cassette *AtRps5a*_*pro*_*Cre*, *AtUbq3*_*pro*_*lox* (from vector pZZ031B, Table S1), or *AtUbq10*_*pro*_*lox* were observed for DsRed/tdTomato expression and counted. If fluorescence was not evident in dry seeds, a proportion of T1 seeds from those lines was germinated between sheets of filter paper saturated with sterile RODI water and observed for DsRed/tdTomato fluorescence after 5 days. For the cowpea lines harboring the cassette *AtDD45*_*pro*_*Cre*, the presence or absence of the transgene in T1 progeny was determined by PCR using genomic DNA extracted from leaf tissues of 2-week-old T1 seedlings grown in the greenhouse. The segregation ratios of the transgene in tobacco and cowpea transgenic lines were calculated and tested by the Chi-square test.

### Estimation of copy number by quantitative PCR (qPCR)

Genomic DNA from transgenic plants harboring the cassette *AtRps5a*_*pro*_*Cre* or *AtUbq3*_*pro*_*lox* was digested by restriction endonuclease *Hin*dIII-HF^®^ (New England Biolabs, Ipswich, MA, USA) while genomic DNA from transgenic plants harboring the cassette *AtDD45*_*pro*_*Cre* or *AtUbq10*_*pro*_*lox* was digested by *Nco*I-HF^®^ (New England Biolabs). After purification with the DNA Clean and Concentration-5 Kit (Zymo Research, Irvine, CA, USA), digested DNAs in 20-fold dilution served as the qPCR templates. The qPCR reactions were conducted with the SYBR green I/HRM dye program in a LightCycler^®^ 480 system (Roche, Basel, Switzerland) following the manufacturer’s protocol of LightCycler 480 SYBR Green I Master V13 (Roche, Mannheim, Germany) in a 20 µl volume. The amplification efficiency of each primer combination was estimated based on qPCR data of a 5-log serial dilution (0.0016, 0.008, 0.04, 0.2, 1×) of a DNA mixture that contained an equal amount of DNA from each tested sample using the absolute quantification method in the LightCycler 480 software (Roche, Release 1.5.0). The qPCR results with two technical replicates for each sample were analyzed using the advanced relative quantification method in the LightCycler 480 software. Primers for qPCR were designed using either Geneious R10 (Auckland, New Zealand) or Realtime PCR Tool from Integrated DNA Technology (San Jose, CA, USA) (Table S4). The tobacco *tubulin α-chain* gene (NCBI accession # XM_016623993) served as a reference gene (Głowacka et al. 2016) for the assays in tobacco. To identify single-copy genes used as reference genes for cowpea, the Plants Datasets (Embryophyta odb9) from Benchmarking Universal Single-Copy Orthologs (BUSCO) was aligned to the whole-genome shot-gun (WGS) assembly (MATU00000000 at NCBI) of cowpea IT97K-499-35 (Muñoz-Amatriaín et al. 2017) using the BUSCO v3 software (Simão et al. 2015; Waterhouse et al. 2018). The amino acid sequences of those orthologs classified as “complete” BUSCO genes were aligned to the reference protein database at NCBI using the BLASTP program to acquire the annotation of the genes. After removing mitochondrial and chloroplastic genes, the cowpea nucleotide sequences which encode the amino acid sequences were aligned to the cowpea WGS assembly to verify whether the gene sequence was unique in the assembly. The genes without any duplication in the genome were considered as single-copy genes in the cowpea genome and potentially used as reference genes for qPCR. After testing the amplification efficiency and specificity of the first six genes from the list, a predicted cowpea *F-box protein* (*VuFbox*, Phytozome12 transcript name Vigun07g146600) was selected and used as the reference gene for the assays in cowpea. The copy numbers of the transgene were calculated based on the ratios of the transgene to the reference gene.

### Southern blot

Genomic DNA of 10 µg from the cowpea lines of the cassettes *AtRps5a*_*pro*_*Cre* and *AtUbq3*_*pro*_*lox* was digested overnight by *Spe*I-HF^®^ and *Mlu*I-HF^®^ (New England Biolabs), respectively. Restriction fragments were separated by gel electrophoresis and then transferred to GeneScreen Plus^®^ hybridization transfer membrane (Cat# NEF1017001PK, PerkinElmer, Waltham, MA, USA). Hybridization was conducted using a digoxigenin (DIG)-labeled probe targeting either the *Cre* gene for the cassette *AtRps5a*_*pro*_*Cre* or the *ZsGreen* gene for the cassette *AtUbq3*_*pro*_*lox* and using DIG Easy Hyb^TM^ as hybridization buffer following the Roche DIG application manual for filter hybridization (Eisel et al. 2008). The probes were generated and labeled by PCR with primers p3769/p3770 and p3700/p3701 (Table S3) for *Cre* and *ZsGreen*, respectively using the Roche PCR DIG labeling mix following the manufacturer’s protocol. Detection was conducted using the chromogenic assay with NBT/BCIP according to the Roche DIG application manual for filter hybridization (Eisel et al. 2008). All chemicals used for making buffers, as well as blocking reagent (Cat# 11096176001), anti-DIG-AP (Cat# 11093274910), and NBT/BCIP (Cat# 11681451001), were from Millipore Sigma.

### Identification of cowpea plants homozygous for the transgenes

The ideal plant materials for evaluating Cre/*lox*-mediated gene activation upon crossing were homozygous plants derived from lines where the transgene segregated as a single locus. For the cassette *AtRps5a*_*pro*_*Cre*, at least six transgenic T1 seedlings from the single-locus lines were grown in the greenhouse to set seeds, and their T2 seeds were observed for *DsRed* expression. If all T2 seeds harvested displayed DsRed fluorescence, the T1 plants were homozygous for the transgene. For the cassettes *AtUbq3*_*pro*_*lox, AtDD45*_*pro*_*Cre* and *AtUbq10*_*pro*_*lox*, the homozygotes were identified using qPCR (Sect. “Estimation of copy number by quantitative PCR (qPCR)”) from the single-locus lines. If the ratio of the transgene to the reference gene in the T1 progeny doubled compared to the T0 mother plants, the T1 progeny would be homozygous for the transgene. Expression of *DsRed/tdTomato* in T2 seeds from the homozygous T1 of the *AtUbq3*_*pro*_*lox* and *AtUbq10*_*pro*_*lox* lines also was observed to verify their homozygosity. The homozygous plants were grown in the greenhouse to increase seeds and make crosses.

We also attempted to identify homozygous progeny that inherited a single locus from the *AtRps5a*_*pro*_*Cre* or *AtUbq3*_*pro*_*lox* lines with multiple copies of the transgene. The numbers of cowpea transgenic lines for the cassettes *AtRps5a*_*pro*_*Cre* and *AtUbq3*_*pro*_*lox* were low due to the low efficiency of the transformation methods applied. To include additional lines harboring those cassettes in the analysis, it was necessary to utilize high copy-number lines. Since the transgenes segregated in the T1 generation, progeny inheriting only one of the transgene loci from those lines could be obtained if enough progeny were screened by qPCR, T1 progeny that likely carried two copies of the transgene were identified and grown in the greenhouse to set seeds. If all the T2 seeds from a T1 displayed DsRed fluorescence, the T1 progeny had become homozygous for the single locus inherited from the T0 parents. If the fluorescence trait segregated among T2 seeds from a T1 progeny, the segregation ratio would indicate the number of transgene loci inherited by the T1 progeny. The T1 progeny with an approximately 3:1 segregation ratio were further advanced to obtain homozygous plants using the same approach as previously described.

### Tobacco crossing and F_***1***_ embryo isolation

Flowers were emasculated at stage 10 (Koltunow et al. 1990) and covered with a tailored pollination envelope. After 2 days, the emasculated flowers on the *AtRps5a*_*pro*_*Cre* lines were pollinated with pollen from the *AtUbq3*_*pro*_*lox* line. Meanwhile, the *AtRps5a*_*pro*_*Cre* lines and the *AtUbq3*_*pro*_*lox* line were self-pollinated as controls. The pollinated flowers were tagged and covered with tailored pollination envelopes. Ovules were harvested 6–8 days after pollination (DAP), and embryos were isolated using the method described by Fu et al. (1996) with some modifications (Zhang et al. 2020). Isolated embryos were observed for *ZsGreen* expression.

### Cowpea crossing and F_1_ progeny analysis

Cowpea flowers were emasculated in the evening before anthesis, followed by covering the peduncles of emasculated flowers with moist tailored pollination envelopes. The next morning, emasculated flowers were pollinated and tagged, and the peduncles of the pollinated flowers were covered by moist tailored pollination envelopes. After harvest, mature F_1_ seeds were observed for *ZsGreen* expression. Genomic DNA was extracted from leaf tissues of F_1_ seedlings. F_1_ progeny were genotyped by PCR to verify whether they inherited both the *Cre* and *lox*-reporter genes. With the use of p3785/p3786 and p4825/p4826, PCR could also confirm the Cre*/lox*-mediated excision in F_1_ progeny inheriting both the *Cre* and *lox*-reporter genes, as the excision would result in a smaller amplicon due to the removal of the intervening DNA sequence flanked by two *lox* sites (Fig. 1b, c).

### Observation of fluorescence

Expression of fluorescent marker genes (i.e., *ZsGreen*, *DsRed*, and *tdTomato*) in plant tissues was observed using a Stemi SVII stereomicroscope equipped with an HBO illuminator (Zeiss, Thornwood, NY, USA), a FITC filter set (λ_excitation_ = 480 nm, and λ_emission_ = 515 nm; Chroma Technology, Bellows Falls, VT, USA), and a DsRED filter (λ_excitation_ = 545/25 nm, dichroic 565LP, λ_emission_ = 605/70 nm; Chroma Technology). Expression of *ZsGreen* in isolated embryos was observed under a Zeiss Axioskop 2 *plus* fluorescence microscope equipped with an 89 North^®^ PhotoFluor LM-75 illumination system (Chroma Technology) and a FITC filter set. Images were taken using an AxioCam camera (Carl Zeiss, Oberkochen, Germany) and the AxioVision LE64 software.

### Nomenclature of transgenic cowpea

We gave a unique code to every T0 transgenic cowpea plant recovered from tissue culture. For the *AtRps5a*_*pro*_*Cre* and *AtUbq3*_*pro*_*lox*, each plant was named by a six-digit code. The first two digits were the last two digits of the vector used; the middle two digits indicated the independent explant giving rise to transgenic shoots (i.e., independent lines); and the last two digits indicated the plants recovered from the particular line. In the code “120200”, for example, ‘12’ indicated the use of vector pZZ012; ‘02’ indicated the independent line 2; and ‘00’ indicated the first plant recovered from line 1202. For the *AtDD45*_*pro*_*Cre* and *AtUbq10*_*pro*_*lox*, each plant was named by a code starting with “R” followed by six digits. In the code “R771402”, for example, ‘77’ indicated the use of vector RC2677; ‘14’ indicated the independent line 14; and ‘02’ indicated the second plant recovered from line R7714. Regardless of the last two digits, as long as the first four digits were the same, the plants were potentially clones from the same line unless additional evidence showed that they were not.

## Results

### Transient Cre/lox-mediated gene activation

**Fig. 2:**
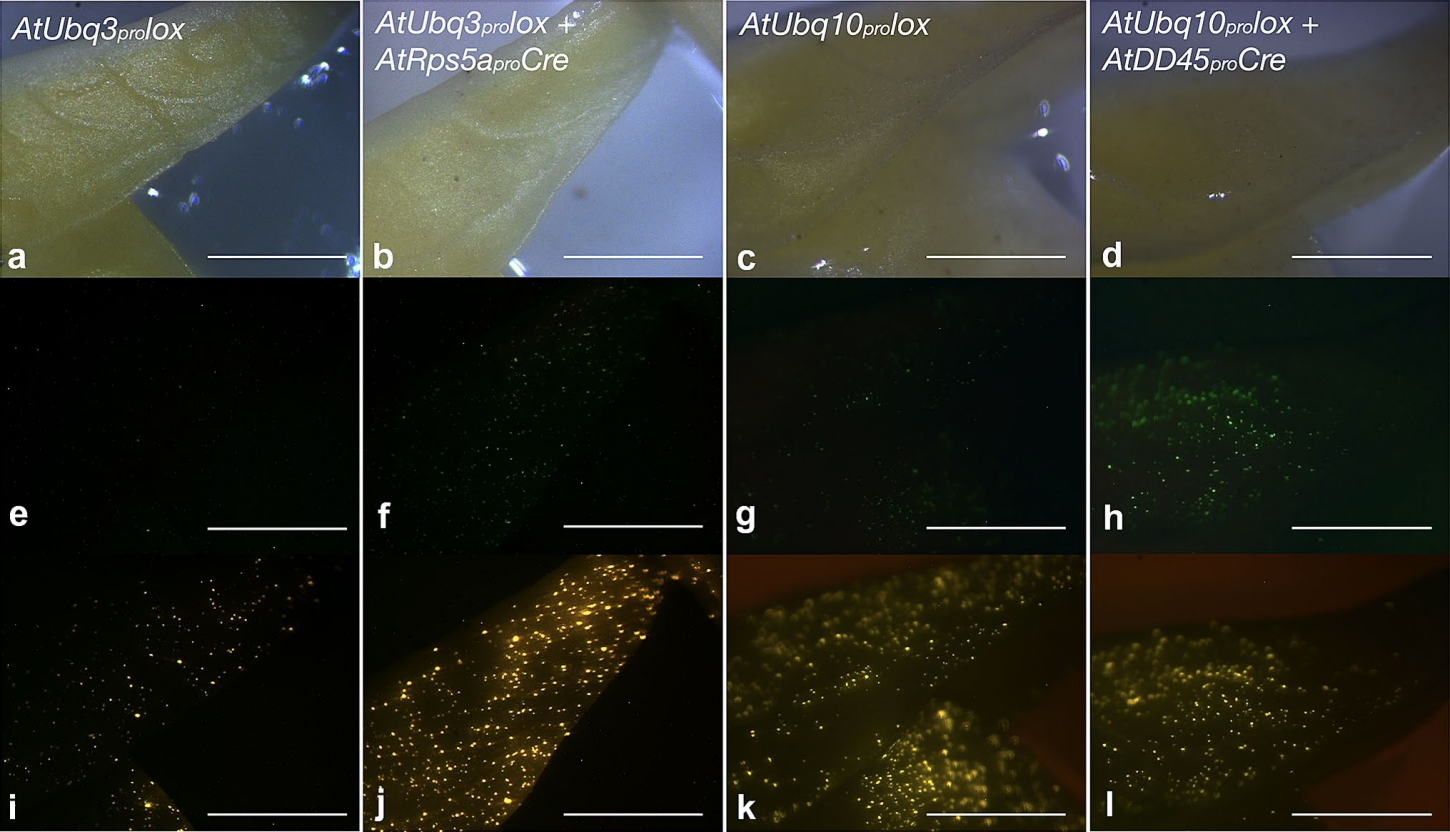
Gene activation via Cre/*lox*-mediated recombination by microprojectile bombardment. Each column displays primary leaves of a cowpea embryo axis 24 h after bombardment under bright field (top), FITC filter (middle), and DsRED filter (bottom). **e**–**h** *ZsGreen* expression indicates activation of the reporter gene; **i**, **j**, *DsRed* expression as an internal control indicates the success of the bombardment; **k**, **l**, *tdTomato* expression from the *lox*-flanked cassette; bar = 1 mm

To validate whether the *Cre* and *lox*-reporter cassettes were functional in cowpea before creating stable transformants, the *Cre* and *lox*-reporter cassettes were co-introduced into cowpea embryo axes by microprojectile bombardment. Upon co-bombardment with the cassettes *AtRps5a*_*pro*_*Cre* and *AtUbq3*_*pro*_*lox*, cowpea embryo axes displayed *ZsGreen* expression 24 h after bombardment (Fig. 2), though the intensity of *ZsGreen* expression was weaker than expected. In preliminary experiments, we observed a stronger expression of *ZsGreen* when cowpea tissues were bombarded by a cassette with the *AtUbq3*_*pro*_ directly followed by the *ZsGreen* CDS and a cassette with a modified *lox* site (see Sect. “*Cre* and *lox*‑reporter gene constructs”, Fig. 1b) present between the *AtUbq3*_*pro*_ and the *ZsGreen* CDS (i.e., the product of treating the cassette *AtUbq3*_*pro*_*lox* with Cre recombinase in vitro) (Fig. S1). Upon co-bombardment with the cassettes *AtDD45*_*pro*_*Cre* and *AtUbq10*_*pro*_*lox*, *ZsGreen* expression was also observed in cowpea embryo axes in 24 h (Fig. 2h). The tissue specificity of the *AtDD45*_*pro*_ might relax under the condition of bombardment. Compared with the cassette *AtUbq3*_*pro*_*lox*, the reporter gene *ZsGreen* in the cassette *AtUbq10*_*pro*_*lox* appeared to have leaky expression, but the bright ZsGreen fluorescence after activation was distinguishable from the leaky expression (Fig. 2).

### Transgenic tobacco

#### Recovery of transgenic tobacco

We introduced the cassettes *AtRps5a*_*pro*_*Cre* and *AtUbq3*_*pro*_*lox* into tobacco and first evaluated their efficiency for Cre/*lox*-mediated gene activation by making crosses in tobacco prior to recovery of transgenic cowpea. Seven independent lines of the cassette *AtRps5a*_*pro*_*Cre* and three independent lines of the cassette *AtUbq3*_*pro*_*lox* were recovered, showing no visual abnormality in plant development compared with non-transgenic. Upon genotyping with primers p3753/p3768, all *AtRps5a*_*pro*_*Cre* lines carried the full-length CDS of the *Cre* gene and the *phaseolin* terminator. Expression of *Cre* was detected in ovules of unpollinated flowers (data not shown). Upon genotyping with primers p3785/p3908, only one *AtUbq3*_*pro*_*lox* line harbored the full-length CDS of the *ZsGreen* gene and the *NOS* terminator without truncation. Based on the *DsRed* expression among T1 progeny from the *AtRps5a*_*pro*_*Cre* T0 lines, the transgene segregated in a 3:1 ratio as a single locus in two lines (pZZ010_1.1 and pZZ010_202.3), in a 15:1 ratio as two independent loci in four lines (pZZ010_2.2, pZZ010_201.2, pZZ012_1.4, and pZZ012_2.1), and likely as four independent loci or more in one line (pZZ010_1.3) (Table S5). Based on the response to kanamycin selection among T1 progeny from the *AtUbq3*_*pro*_*lox* line (pZZ017_1.1) carrying the complete cassette, the transgene likely segregated as four independent loci (Table S5). Based on the phenotype of T2 progeny, we identified homozygous T1 progeny from two single-locus *AtRps5a*_*pro*_*Cre* lines where all T2 progeny displayed DsRed fluorescence and resistance to hygromycin selection (Table S6).

#### Cre/lox-mediated gene activation in tobacco by crossing

**Fig. 3:**
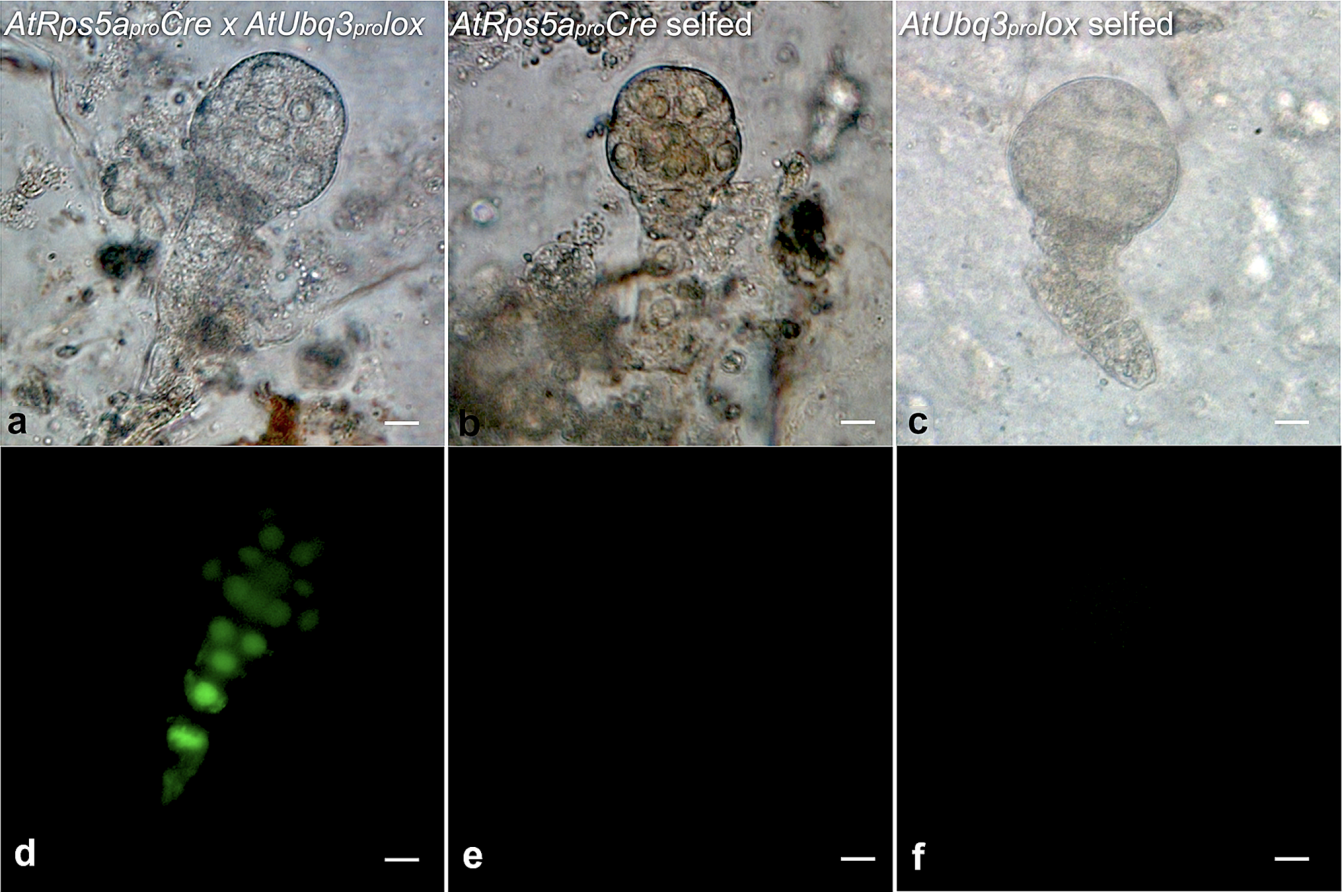
Cre/*lox*-mediated gene activation in isolated F_1_ tobacco embryos after crosses between the *AtRps5a*_*pro*_*Cre* line and the *AtUbq3*_*pro*_*lox* line. **a**–**c** Bright field, **d**–**f** FITC filter; bar = 10 µm

**Fig. 4:**
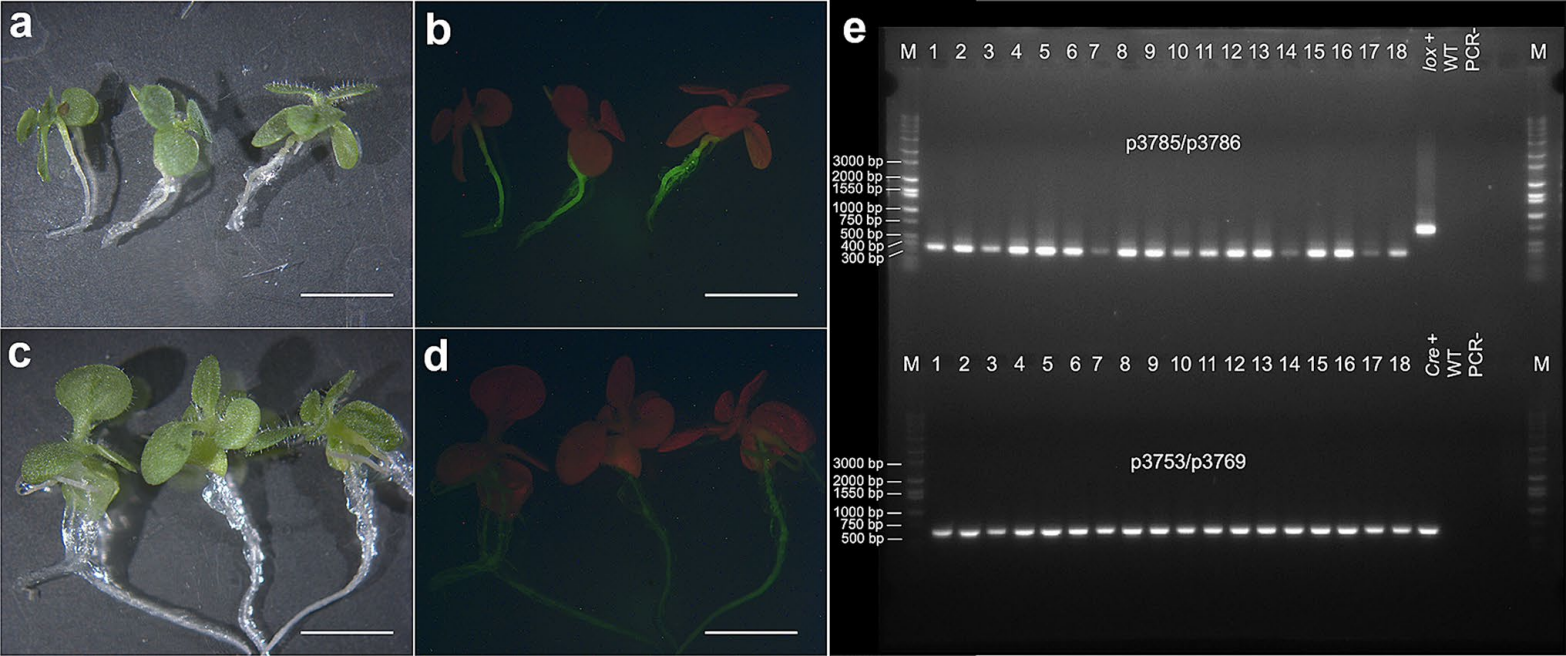
Cre/*lox*-mediated recombination in F_1_ progeny of tobacco from a cross between the *AtRps5a*_*pro*_*Cre* line and the *AtUbq3*_*pro*_*lox* line. **a**, **b** F_1_ seedlings from a cross between a homozygous plant of the *AtRps5a*_*pro*_*Cre* line and a T1 plant of the *AtUbq3*_*pro*_*lox* line; **c**, **d** T2 seedlings of the *AtUbq3*_*pro*_*lox* line; **a**, **c** bright field; **b**, **d** FITC filter; **e** electrophoresis of PCR products with p3785/p3786 (top panel, the *lox*-reporter cassette) and p3753/p3769 (bottom panel, *Cre*); 1–18, F_1_ seedlings randomly sampled; *lox+*, the *AtUbq3*_*pro*_*lox* line; *Cre+*, the *AtRps5a*_*pro*_*Cre* line; *WT* wild type; PCR-, no-template control; M, DNA ladder; bar = 5 mm

With six *AtRps5a*_*pro*_*Cre* T0 lines pollinated by the *AtUbq3*_*pro*_*lox* T0 line (line pZZ017_1.1), *ZsGreen* expression was observed in isolated F_1_ embryos at an early developmental stage (Fig. 3). Activation of *ZsGreen* occurred in F_1_ embryos from crosses with five *AtRps5a*_*pro*_*Cre* lines at a frequency ranging from 12.1% to 82.6% (Table 1). In contrast, self-pollination of the *AtUbq3*_*pro*_*lox* T0 line or *AtRps5a*_*pro*_*Cre* T0 lines resulted in no *ZsGreen*-expressing embryos. The expected ratios of ZsGreen-positive to ZsGreen-negative embryos were calculated based on the assumption that the transgenes in all loci were functional and excision occurred at 100% (Table 1). Expression of *ZsGreen* segregated as expected in F_1_ embryos of three lines, while the number of *ZsGreen*-positive F_1_ embryos was lower than expected in line pZZ010_1.1 and pZZ010_201.2 (Table 1). With the homozygous T1 progeny of a single-locus *AtRps5a*_*pro*_*Cre* line (pZZ010_1.1) pollinated by T1 progeny of the *AtUbq3*_*pro*_*lox* line pZZ017_1.1, the frequency of isolated *ZsGreen*-expressing F_1_ embryos varied from 7.1% to 70.0% (Table S7). Without identifying progeny of the *AtUbq3*_*pro*_*lox* line homozygous for all transgene loci, we could not know whether an isolated embryo inherited both the *Cre* and *lox*-reporter cassette. Therefore, we decided to estimate the efficiency of Cre/*lox*-mediated gene activation based on the *ZsGreen* expression in F_1_ seedlings rather than isolated F_1_ embryos, since the F_1_ seedlings could be tested for both transgenes by genotyping. When F_1_ seeds from crosses made between the homozygous T2 plants of line pZZ010_1.1 and the T2 plants of the *AtUbq3*_*pro*_*lox* T0 line pZZ017_1.1 germinated, strong *ZsGreen* expression was not observed as expected in any F_1_ seedlings and dim ZsGreen fluorescence was detected only in root tissues (Fig. 4). However, genotyping by PCR indicated removal of the *PINII*_*term*_ in F_1_ seedlings inheriting both the *Cre* and the *lox*-reporter cassette given the amplification of a 333-bp fragment rather than a 699-bp one with p3785/p3786 (Fig. 4e). Upon searching for the expression profile of *AtUbq3* (AT5G03240) visualized on the *Arabidopsis* eFP Browser (Schmid et al. 2005; Winter et al. 2007), we found that the *AtUbq3*_*pro*_ drives consistently low gene expression across many tissues, except for dry seeds, the second internode, and mature pollen with a relatively higher expression (Fig. S2). The difficulty to detect ZsGreen fluorescence in F_1_ seedlings could be attributable to the low expression of *ZsGreen* driven by the *AtUbq3*_*pro*_. Genotyping results on a larger scale showed that all F_1_ seedlings inheriting both transgenes yielded only the 333 bp amplicon, indicating complete removal of *PINII*_*term*_ by Cre*/lox*-mediated recombination at 100% across samples and the unlikeliness of mosaic F_1_ progeny (Table 2).

**Table 1:** Cre/*lox*-mediated gene activation in F_1_ embryos from crosses of T0 tobacco lines

Female parent (*AtRps5a*_*pro*_*Cre*)	Male parent (*AtUbq3*_*pro*_*lox*)	# ZsGreen+ embryos	# ZsGreen− embryo	# Total embryos observed	% ZsGreen+ embryos	Segregation ratio (*p*-value^a^)
pZZ010_1.1	pZZ017_1.1	12	41	53	22.6%	15:17 (<0.001)
pZZ010_1.3	pZZ017_1.1	57	12	69	82.6%	225:31 (0.179)
pZZ010_2.2	pZZ017_1.1	45	12	57	78.9%	45:19 (0.154)
pZZ010_201.2	pZZ017_1.1	7	51	58	12.1%	45:19 (<0.001)
pZZ010_202.3	pZZ017_1.1	0	60	60	0	15:17 (<0.001)
pZZ012_1.4	pZZ017_1.1	29	22	51	56.9%	45:19 (0.036)

^a^Segregation ratios were tested by Chi-square

**Table 2:** Cre/*lox*-mediated excision in F_1_ progeny of tobacco from crosses with homozygous T2 *AtRps5a*_*pro*_*Cre* lines pollinated by T2 *AtUbq3*_*pro*_*loxP* lines

Female parent (*AtRps5a*_*pro*_*Cre*)	Male parent (*AtUbq3*_*pro*_*lox*)	# F_1_ seedlings with the *Cre* & *lox*-reporter genes	# F_1_ seedlings with *PINII*_*term*_ excised	Excision efficiency (*PINII*_*term*_–/*Cre+* & *lox+*)
pZZ010_1.1_1_1	pZZ017_1.1_8.1_4	62	62	100%
pZZ010_1.1_1_1	pZZ017_1.1_4.2_4	55	55	100%
pZZ010_202.3_11_1	pZZ017_1.1_4.2_4	63	63	100%
pZZ010_202.3_11_1	pZZ017_1.1_8.1_2	78	78	100%

### Transgenic cowpea

#### Recovery of transgenic cowpea

When the cassettes *AtRps5a*_*pro*_*Cre* and *AtUbq3*_*pro*_*lox* were introduced into cowpea using Method 1 and 2, transgenic plant recovery was very inefficient. For the cassette *AtRps5a*_*pro*_*Cre*, three independent lines (out of 464 explants) and one transgenic line (out of 489 explants) were recovered through kanamycin or PPT selection, respectively. No transgenic plant (out of 481 explants) was recovered through hygromycin selection. All transgenic lines harbored the full-length CDS of *Cre* and the *phaseolin* terminator as determined by genotyping with primers p3753/p3768. According to qPCR, one line (1201) had a single copy of the transgene, one line (1101) likely had two copies, one line (1202) had four copies, and one line (1203) likely had 6-8 copies of the transgene (Table S8). Southern blot analysis verified that line 1201 had one transgene copy, line 1101 had two copies, and lines 1202 and 1203 had multiple copies that were too complex to separate by gel electrophoresis (data not shown). The consistency between qPCR and Southern blot suggested that the designed qPCR assay could effectively estimate the copy number of the transgene, at least for the low copy-number lines; therefore, qPCR was used for the rest of the study. Based on the expression of *DsRed* among T1 seeds, the transgene segregated in a 3:1 ratio as a single locus in the *AtRps5a*_*pro*_*Cre* lines 1101 and 1201 (Table S9). For the cassette *AtUbq3*_*pro*_*lox*, 2 independent lines (out of 456 explants) were recovered through kanamycin selection, and both harbored the full-length CDS of the *ZsGreen* and the terminator as shown by genotyping (primers p3785/p3908). According to qPCR, one line (3102) contained a single copy of the transgene while the other line (3101) likely contained six copies.

When the cassettes *AtDD45*_*pro*_*Cre* and *AtUbq10*_*pro*_*lox* were introduced into cowpea using Method 3 with spectinomycin selection, recovery of transgenic plants was much more efficient. Nine lines (out of 271 explants) and 13 lines (out of 271 explants) were recovered for these 2 cassettes, respectively, (Table S8) without the use of *in vitro* micrografting. All transgenic lines harbored the full-length CDS of the transgene and the terminator based on PCR genotyping. According to qPCR for the *AtDD45*_*pro*_*Cre* lines, one line (R7719) had a single copy of the transgene, one line (R7743) had 1-2 copies, one line (R7715) had 2-3 copies, and the other six lines had at least three copies (Table S8). As line R7719 senesced quickly after entering the reproductive stage and produced only one pod with one non-transgenic seed capable of germination, we could not maintain this single-copy *AtDD45*_*pro*_*Cre* line for further analysis. The transgene segregated as a single locus for several lines, although they harbored more than one copy of the transgene. When T1 seedlings of lines R7743, R7714, and R7715 were genotyped with primers p4261/p4262, transgene segregation fit a 3:1 ratio indicating a single locus in these three lines (Table S10). According to qPCR for the *AtUbq10*_*pro*_*lox* lines, eight lines (R170102, R170103, R1702, R1713, R1714, R1716, R1720, and R1739) had 1-2 copies of the transgene, three lines (R1704, R1719, and R1744) had 2-3 copies and the other two lines (R1723 and R1725) had at least three copies (Table S8). Twelve *AtUbq10*_*pro*_*lox* lines were capable of producing T1 seeds except for line R1744. Since the expression of *tdTomato* in mature T1 seeds of the *AtUbq10*_*pro*_*lox* lines was relatively weak except for line R1702, the transgene segregation ratio of these lines based on tdTomato fluorescence was calculated from seedlings instead of dry seeds. Based on the expression of *tdTomato* in T1 progeny of seven lines, the transgene segregated in a 3:1 ratio in lines R1702, R1719, and R1739 that carried at least two copies of the transgene (Table S10). The transgene segregated in a 15:1 ratio as two independent loci in lines R170103 and R1714. Segregation distortion was found in lines R170102, R1713, and R1720 (Table S10), and they likely carried 1-2 copies of the transgene according to the qPCR results (Table S8).

#### Identifying homozygous transgenic cowpea plants for making genetic crosses

Homozygous progeny of the lines with a simple transgene integration are preferable to evaluate Cre/*lox*-mediated gene activation upon crossing. Homozygous T1 progeny of the two single-locus *AtRps5a*_*pro*_*Cre* lines (1101 and 1201) were identified based on the expression of *DsRed* in T2 seeds (Table S11). By qPCR, we identified homozygous T1 progeny of the *AtUbq3*_*pro*_*lox* line 3102 (Table S12), three single-locus *AtDD45*_*pro*_*Cre* lines (R7714, R7715, and R7743), and three single-locus *AtUbq10*_*pro*_*lox* lines (R1702, R1719, and R1719) (Table S13).

Since only a few transgenic lines were recovered for the cassette *AtRps5a*_*pro*_*Cre*, T1 progeny that inherited a single transgene locus from the high-copy transgenic lines were identified to evaluate Cre/*lox*-mediated gene activation with the *Cre* expressed by the *AtRps5a*_*pro*_. T1 transgenic progeny of lines 1202 and 1203 that likely inherited two copies of the transgene *AtRps5a*_*pro*_*Cre* were identified based on qPCR, and a homozygous plant of line 1203 was identified based on expression of *DsRed* in T2 seeds (Table S14).

#### Cre/lox-mediated gene activation in cowpea by crossing

**Fig. 5:**
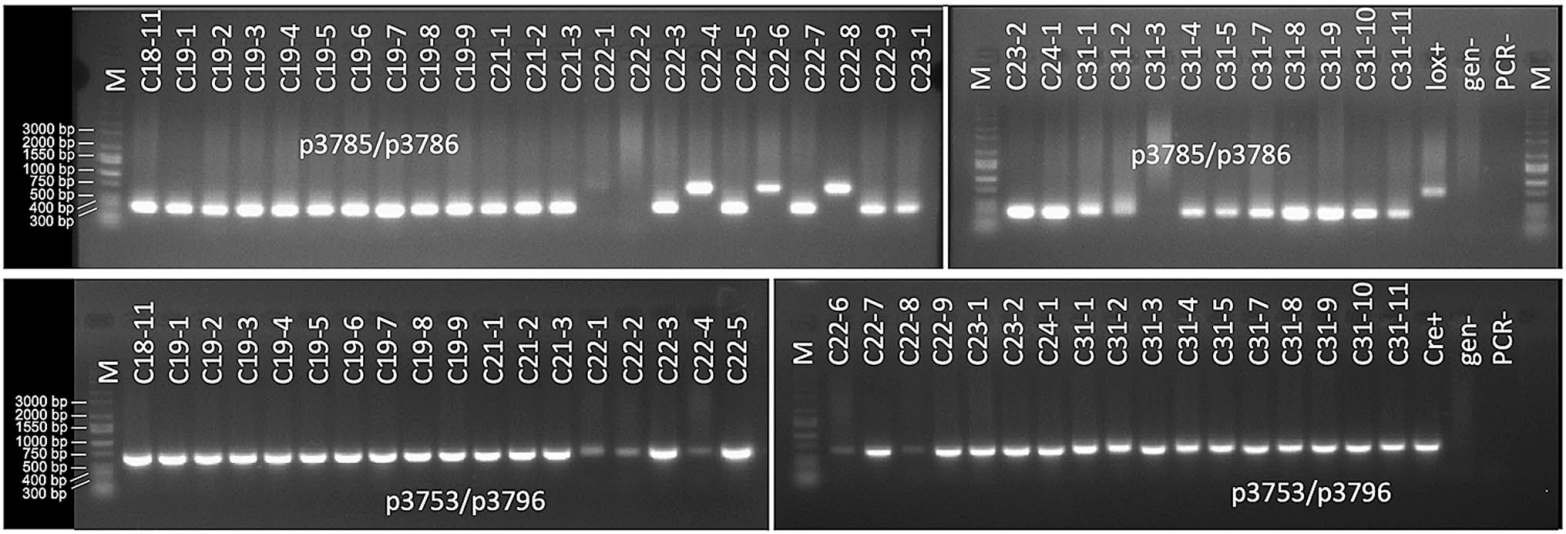
An example of electrophoresis of PCR amplicons with p3785/p3786 (top, the *lox*-reporter cassette) and p3753/p3796 (bottom, *Cre*) for cowpea F_1_ progeny of crosses between homozygous plants of the *AtRps5a*_*pro*_*Cre* lines and an *AtUbq3*_*pro*_*loxP* line. C18-11 was the 11th seedling of cross 18 (line 3102 × line 1201); C19-1 to C19-9 were the nine seedlings of cross 19 (line 3102 × line 1201); C21-1 to C21-3 were the three seedlings of cross 21 (line 3102 × line 1203); C22-1 to C22-9 were nine seedlings of cross 22 (line 3102 × line 1203); C23-1 to C23-2 were two seedlings of cross 23 (line 3102 × line 1203); C24-1 was the only seedling of cross 24 (line 3102 × line 1203); C31-1 to C31-11 were ten seedlings of cross 31 (line 1203 × line 3102); *lox*+, the *AtUbq3*_*pro*_*lox* line; *cre+*, the *AtRps5a*_*pro*_*Cre* line; gen−, non-transgenic IT86D-1010; PCR−, no-template control for PCR; M, DNA ladder

Gene activation by Cre/*lox*-mediated recombination was first evaluated in the crosses between the *AtRps5a*_*pro*_*Cre* lines and the *AtUbq3*_*pro*_*lox* lines. Similar to the observation in tobacco, ZsGreen fluorescence was probably too weak to detect in the F_1_ mature seeds or any tissues of the seedlings. The F_1_ progeny inheriting both transgenes showed excision of the *PINII*_*term*_, as evidenced by the predicted smaller amplicon (Fig. 5). *ZsGreen* expression was observed in pods during the maturation process as well as the immature F_2_ seeds from the F_1_ plants with the *PINII*_*term*_ excised (Fig. S3). The ZsGreen fluorescence was undetected in younger pods, dry pods, or dry seeds (data not shown). *ZsGreen* expression was not observed in any other tissues except for the peduncle tip when it whitened (Fig. S4). These observations suggested that the difficulty to observe ZsGreen fluorescence in dry seeds and seedlings was possibly due to the low activity of the *AtUbq3*_*pro*_ in these tissues.

Although we were unable to evaluate gene activation by screening for *ZsGreen* expression in F_1_ seeds or seedlings from the crosses with the *AtUbq3*_*pro*_*lox* lines, the efficiency of Cre/*lox*-mediated excision could be determined by genotyping. The efficiency of excision should theoretically be the same as the efficiency of activating the *lox*-reporter gene if it were visible. Based on genotyping, Cre/*lox*-mediated excision occurred in all F_1_ progeny from crosses between homozygous plants of *AtRps5a*_*pro*_*Cre* lines 1101, 1201, and 1203 pollinated by homozygous plants of *AtUbq3*_*pro*_*lox* line 3102 (Table 3). In their reciprocal crosses, excision also took place in all F_1_ progeny (Table 3), except for a cross between line 3102 and line 1203 where three F_1_ progeny showed the 699-bp instead of the 333 bp amplicon (Fig. 5). With line 3101 pollinated by four *AtRps5a*_*pro*_*Cre* lines, excision occurred in all F_1_ progeny inheriting both transgenes except for one cross (line 3101 pollinated by line 1202) (Table 3) wherein F_1_ progeny (2/6) displayed both the 333-bp and the 699-bp amplicons together. Among five crosses between homozygous progeny of line 3102 and an *AtDD45*_*pro*_*Cre* line (R7743) at the T0 generation (i.e., hemizygous), Cre/*lox*-mediated excision seemed to take place in fewer F_1_ progeny inheriting both transgenes, compared with the crosses between line 3102 and the *AtRps5a*_*pro*_*Cre* lines (Table 3). Although the *AtDD45*_*pro*_ presumably expressed *Cre* in a much shorter time course and more tissue-specifically than *the AtRps5a*_*pro*_, no F_1_ progeny showed both 333-bp and 699-bp amplicons together upon genotyping with p3785/p3786, indicating that a tighter expression of *Cre* did not result in higher mosaicism.

**Table 3:** Cre/*lox*-mediated excision in F_1_ progeny of cowpea from crosses between *AtUbq3*_*pro*_*lox* lines and *Cre* lines

Female parent	Male parent	# crosses (pods)	# F1 seedlings with *Cre* and *lox*-reporter genes	# F1 seedlings with *PINII*_*term*_ excised	Excision efficiency (%)^a^
*AtRps5aproCre* 1101	*AtUbq3prolox* 3102	5	31	31	100 ± 0.0% a
*AtRps5aproCre* 1201	*AtUbq3prolox* 3102	2	14	14	100 ± 0.0% a
*AtRps5aproCre* 1203	*AtUbq3prolox* 3102	3	27	27	100 ± 0.0% a
*AtDD45proCre* R7743	*AtUbq3prolox* 3102	4	13	6	47.9 ± 7.4% b
*AtUbq3prolox* 3102	*AtRps5aproCre* 1201	7	44	44	100 ± 0.0% a
*AtUbq3prolox* 3102	*AtRps5aproCre* 1203	5	16	11	88.9 ± 9.9% a
*AtUbq3prolox* 3102	*AtDD45proCre* R7743	5	26	16	62.0 ± 5.6% b
*AtUbq3prolox* 3101	*AtRps5aproCre* 1101	1	7	7	100 ± 0.0% a
*AtUbq3prolox* 3101	*AtRps5aproCre* 1201	2	11	11	100 ± 0.0% a
*AtUbq3prolox* 3101	*AtRps5aproCre* 1202	4	11	9	91.7 ± 7.2% a
*AtUbq3prolox* 3101	*AtRps5aproCre* 1203	4	13	13	100 ± 0.0% a

^a^Data represent means ± standard error. Mean values followed by different letters are significantly different based on Kruskal–Wallis test (α = 0.05).

To improve the ability to observe fluorescent reporter gene activation in F_1_ seeds or seedlings, we generated another *lox-*reporter cassette (i.e., *AtUbq10*_*pro*_*lox*) with the *ZsGreen* regulated by the *AtUbq10*_*pro*_ after Cre/*lox*-mediated recombination. A pilot experiment with homozygous plants from an *AtRps5a*_*pro*_*Cre* line (1101) pollinated by two *AtUbq10*_*pro*_*lox* lines (R1701 and R1714) at the T0 generation showed that all F_1_ seeds displayed ZsGreen fluorescence if they inherited both transgenes, indicating that gene activation could be visualized in F_1_ seeds with the new *lox*-reporter cassette. Genotyping by PCR with primers p4797/p4798 verified the excision of the *lox*-flanked cassette *GmUbq3*_*pro*_*tdTomato* located in F_1_ progeny, showing an amplicon of 2589 bp rather than 5822 bp (data not shown).

**Fig. 6:**
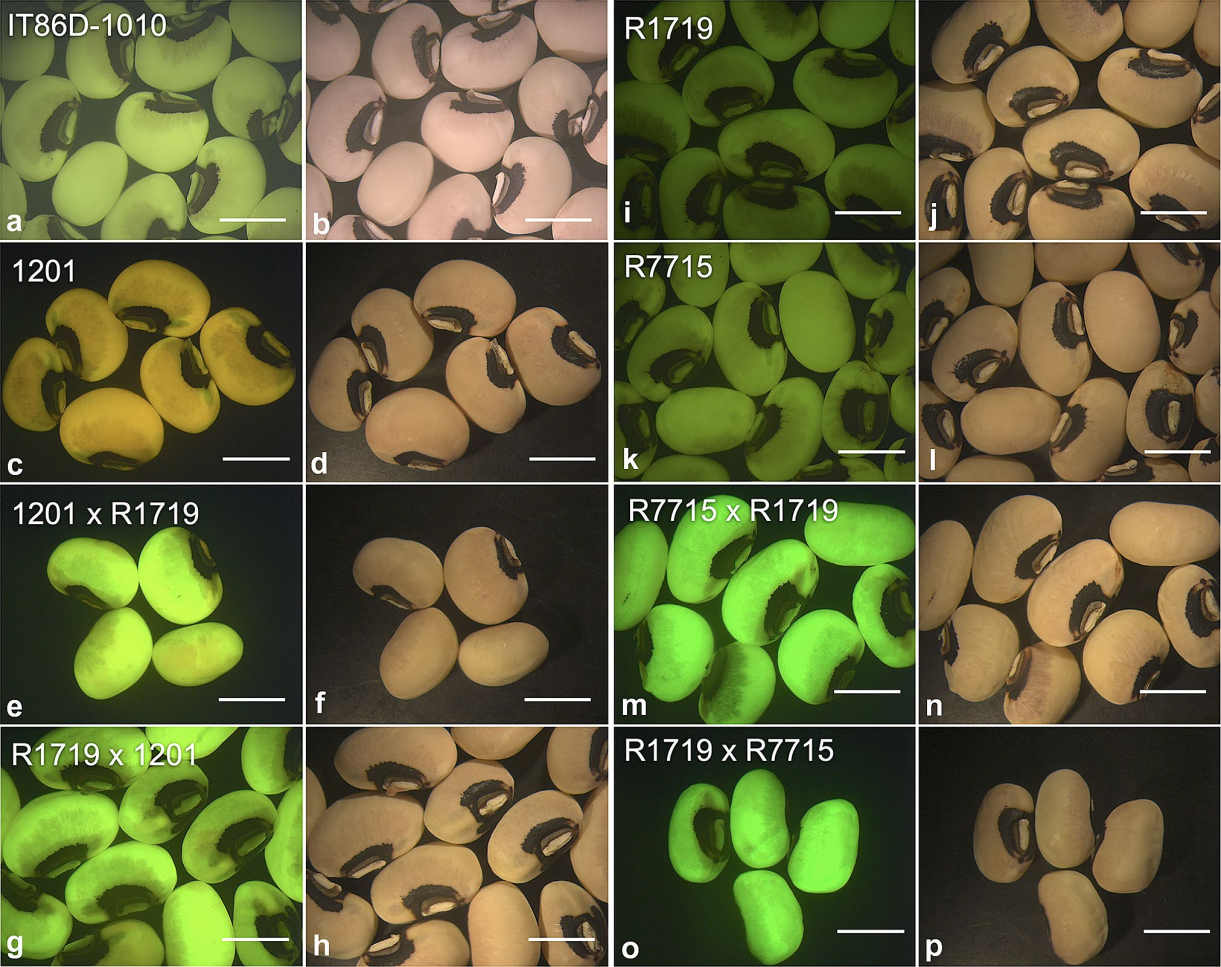
Gene activation via Cre/*lox*-mediated recombination in F_1_ seeds of cowpea from crosses between an *AtUbq10*_*pro*_*lox* line (R1719) and an *AtRps5a*_*pro*_*Cre* line (1201) and an *AtDD45*_*pro*_*Cre* line (R7715). **a**, **c**, **e**, **g**, **i**, **k**, **m**, **o**, FITC filter; **b**, **d**, **f**, **h**, **j**, **l**, **n**, **p**, bright field; bar = 5 mm

**Fig. 7:**
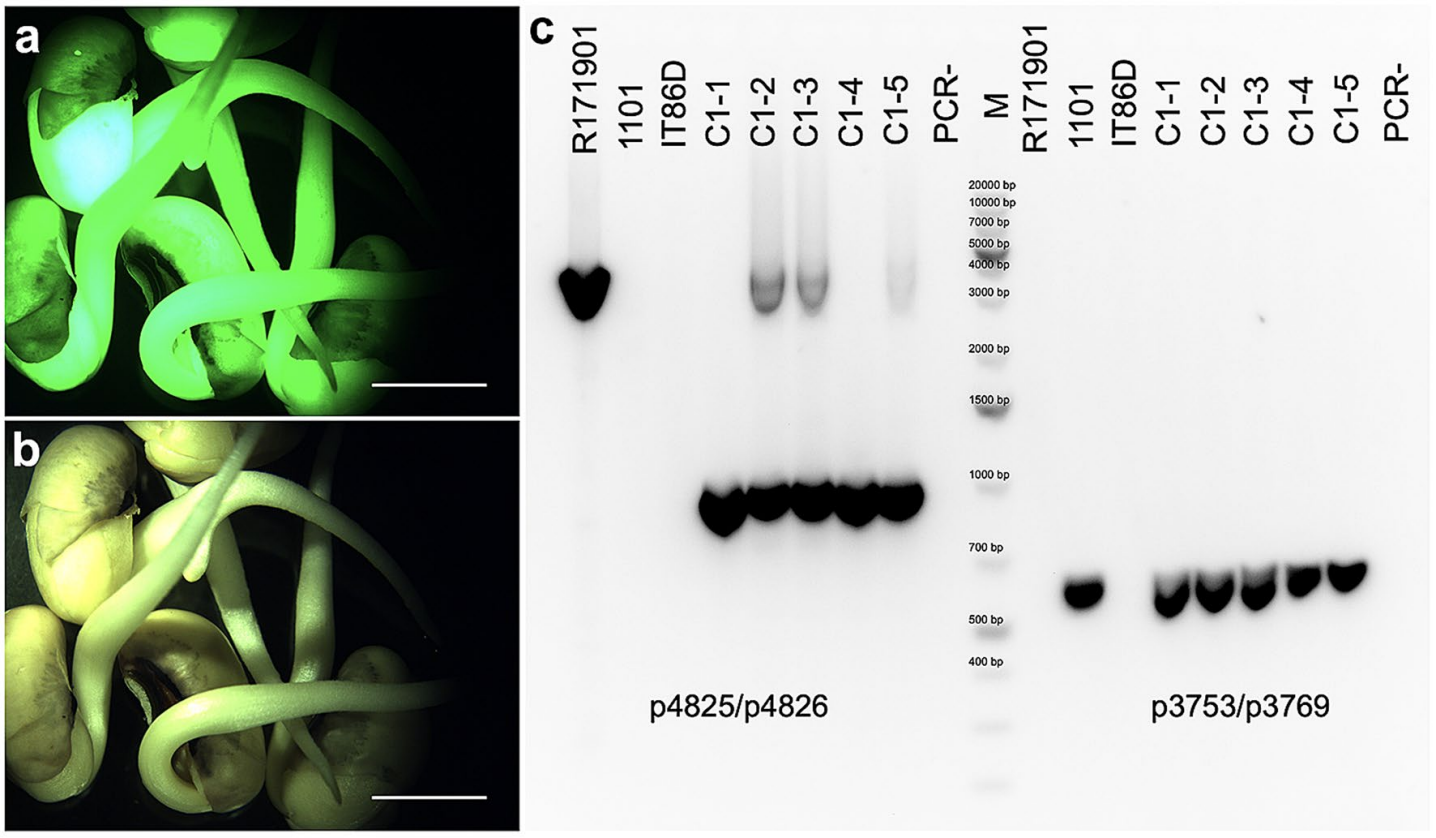
Cre/*lox*-mediated recombination in cowpea F_1_ progeny from a cross between an *AtRps5a*_*pro*_*Cre* line (1101) and an *AtUbq10*_*pro*_*lox* line (R1719). **a**, **b** F_1_ seedlings from a cross between homozygous plants of line 1101 and line R1719; **a** FITC filter; **b** bright field; **c** electrophoresis of PCR products for five F_1_ progeny (C1-1 to C1-5) from the same cross with p4825/p4826 and p3753/p3769; PCR−, no-template control; M, DNA ladder; bar = 5 mm

Among crosses between homozygous progeny of the single-locus *AtUbq10*_*pro*_*lox* lines and the single-locus *AtRps5a*_*pro*_*Cre* or *AtDD45*_*pro*_*Cre* lines, gene activation generally occurred at a comparable frequency. Except for a few crosses, gene activation occurred in all F_1_ progeny from the crosses between the *AtUbq10*_*pro*_*lox* line R1719 and the *Cre* lines (Table 4) based on the *ZsGreen* expression in mature F_1_ seeds (Fig. 6). For three *AtRps5a*_*pro*_*Cre* lines pollinated by line R1719, *ZsGreen* was expressed in over 98% of F_1_ progeny (Table 4). For three *AtDD45*_*pro*_*Cre* lines pollinated by line R1719, *ZsGreen* was expressed in all F_1_ progeny for both lines R7714 and R7715 while less than a half of the F_1_ progeny for line R7743 (Table 4). In the reciprocal crosses with line R1719 pollinated by the *Cre* lines, gene activation occurred in over 83% of F_1_ progeny for two *AtRps5a*_*pro*_*Cre* lines (1201 and 1203) and two *AtDD45*_*pro*_*Cre* lines (R7715 and R7743) (Table 4). With line R1719 pollinated by line R7714, gene activation occurred in 56% of F_1_ progeny in one cross and was undetected in the other one. Strong *ZsGreen*-expression was observed in F_1_ seedlings (Fig. 7a). Upon PCR genotyping with primers p4825/p4826, all *ZsGreen*-expressing seedlings showed 1049 bp amplicon, indicating excision of the *lox*-flanked cassette (Fig. 7c). A weak amplicon of about 4 kb showed in some progeny tested (Fig. 7c) suggesting the presence of the intact *AtUbq10*_*pro*_*lox* cassette without the *lox*-flanked DNA sequence excised. Considering that line 1719 carried 2–3 copies of the transgene (Table S8), Cre/*lox*-mediated excision could fail to occur in every copy of the transgene in those F_1_ progeny but may have taken place in every cell, as none of the F_1_ seedlings appeared to be mosaic by showing a sector without ZsGreen fluorescence (Fig. 7a). All F_1_ progeny from crosses of two *AtRps5a*_*pro*_*Cre* lines (1203 and 1101) with another *AtUbq10*_*pro*_*lox* line (R1702) displayed ZsGreen fluorescence (Table 4), indicating gene activation at a frequency of 100%.

**Table 4:** Cre/*lox*-mediated gene activation in cowpea F_1_ progeny from crosses between homozygous progeny of the *AtUbq10*_*pro*_*lox* lines and homozygous progeny of *Cre* lines.

Female parent	Male parent	# Crosses (pods)	# ZsGreen+ F_1_ seeds	# Total F_1_ seeds	Efficiency of gene activation (%)^a^
*AtRps5a*_*pro*_*Cre* 1201	*AtUbq10*_*pro*_*lox* R1719	5	39	40	98.6 ± 1.4% ab
*AtRps5a*_*pro*_*Cre* 1203	*AtUbq10*_*pro*_*lox* R1719	3	23	23	100.0 ± 0.0% a
*AtRps5a*_*pro*_*Cre* 1101	*AtUbq10*_*pro*_*lox* R1719	4	41	41	100.0 ± 0.0% a
*AtDD45*_*pro*_*Cre* R7743	*AtUbq10*_*pro*_*lox* R1719	3	4	10	42.9 ± 29.7% ab
*AtDD45*_*pro*_*Cre* R7715	*AtUbq10*_*pro*_*lox* R1719	2	16	16	100.0 ± 0.0% a
*AtDD45*_*pro*_*Cre* R7714	*AtUbq10*_*pro*_*lox* R1719	1	5	5	100.0% a
*AtUbq10*_*pro*_*lox* R1719	*AtRps5a*_*pro*_*Cre* 1201	2	16	17	83.3 ± 16.7% ab
*AtUbq10*_*pro*_*lox* R1719	*AtRps5a*_*pro*_*Cre* 1203	4	29	29	100.0 ± 0.0% a
*AtUbq10*_*pro*_*lox* R1719	*AtDD45*_*pro*_*Cre* R7743	3	11	12	83.3 ± 16.7% a
*AtUbq10*_*pro*_*lox* R1719	*AtDD45*_*pro*_*Cre* R7715	4	13	13	100.0 ± 0.0% a
*AtUbq10*_*pro*_*lox* R1719	*AtDD45*_*pro*_*Cre* R7714	2	5	11	27.8 ± 27.8% b
*AtRps5a*_*pro*_*Cre* 1203	*AtUbq10*_*pro*_*lox* R1702	3	14	14	100.0 ± 0.0% a
*AtRps5a*_*pro*_*Cre* 1101	*AtUbq10*_*pro*_*lox* R1702	2	13	13	100.0 ± 0.0% a

^a^Data represent means ± standard error. Mean values followed by different letters are significantly different based on Kruskal–Wallis test (α = 0.05)

## Discussion

This study demonstrates the efficacy of Cre/*lox*-mediated gene activation through genetic crosses in cowpea. Gene activation through Cre/*lox*-mediated excision occurred at a high frequency and probably at an early developmental stage of embryos upon expression of *Cre* by the *AtRps5a*_*pro*_ or *AtDD45*_*pro*_. We first evaluated Cre/*lox*-mediated recombination by sexual hybridization with *Cre* driven by the *AtRps5a*_*pro*_ and the “reporter” gene driven by the *AtUbq3*_*pro*_ after excision of the *lox*-flanked DNA sequence. Because the process of recovering transgenic cowpea was inefficient at the time we introduced these two vectors into cowpea, we also evaluated gene activation with these two vectors in tobacco. The function of Cre/*lox*-mediated recombination has been reported in tobacco with *Cre* expressed by either the *CaMV35S*_*pro*_ (Odell et al. 1990; Bayley et al. 1992; Medberry et al. 1995) or seed-specific promoters (Odell et al. 1994). Compared to those reports with *Cre* expressed by either the *CaMV35S*_*pro*_ or seed-specific promoters, the current study showed that recombination could occur at an earlier developmental stage and higher frequency with *Cre* controlled by the *AtRps5a*_*pro*_. Although we did not obtain direct evidence of gene activation at the zygote stage, gene activation via Cre/*lox*-mediated recombination occurred early in embryo development based on suspensor and globular embryo fluorescence. If the activity of the *AtRps5a*_*pro*_ in tobacco mirrored the expression profile observed in *Arabidopsis* (Weijers et al. 2001; Maruyama et al. 2013; Gooh et al. 2015), *Cre* was expressed in egg cells and young embryos which activated *ZsGreen* early after fertilization. Such results were not achieved by expressing *Cre* with the *CaMV35S*_*pro*_ or seed-specific promoters (Odell et al. 1994). The results confirmed that gene activation via Cre/*lox*-mediated recombination can occur earlier than previously reported if *Cre* is expressed by a promoter having activity in egg cells and young embryos.

Using the *AtRps5a*_*pro*_ to express *Cre* also contributed to the rarity of mosaic F_1_ progeny both in tobacco and cowpea based on the genotyping results of the crosses with the *AtUbq3*_*pro*_*lox* lines. Amplification of both fragments in the F_1_ progeny in a few instances may not necessarily indicate mosaicism. Alternatively, it might reflect that excision did not happen in every copy of the transgene inherited from a parent line that harbored multiple copies of the *AtUbq3*_*pro*_*lox* cassette (Table S8). The absence of mosaicism in most F_1_ progeny was likely ascribed to the expression of *Cre* by the *AtRps5a*_*pro*_ in the egg cells and young embryos. The presence of Cre recombinase in the egg cells ideally would enable the excision of the *lox*-flanked DNA sequence and activation of *ZsGreen* in the zygote that could pass the activated *ZsGreen* to all of its daughter cells. Even if gene activation did not occur in the zygote stage, a strong expression of *Cre* by the *AtRps5a*_*pro*_ during early cell divisions could complete the activation process quickly in the majority of cells when the embryos only consisted of a few cells. Although the *AtRps5a*_*pro*_ activity would be inclined to early activation of the *lox*-reporter gene, we never observed *ZsGreen* expression in all isolated tobacco embryos from any crosses with the *AtRps5a*_*pro*_*Cre* lines pollinated by the *AtUbq3*_*pro*_*lox* line (Table 1, S7). Without identifying homozygous plants of the *AtUbq3*_*pro*_*lox* line carrying multiple transgene loci, we could not determine whether the absence of *ZsGreen* expression in the isolated F_1_ embryos was attributable to inactivation of the reporter gene or the lack of the *lox*-reporter cassette. Considering that the *AtRps5a*_*pro*_ remains active in embryos after the globular stage and even in the meristematic regions during later plant development (Weijers et al. 2001), we cannot exclude the possibility that gene activation might occur at later developmental stages. Such a pattern was observed in gene editing by CRISPR/Cas9 (clustered regularly interspaced short palindromic repeats/CRISPR-associated 9) in *A. thaliana* with the Cas9 expressed by the *AtRps5a*_*pro*_ (Tsutsui and Higashiyama 2017). The activity of the *AtRps5a*_*pro*_ at later developmental stages likely caused heterogeneity in mutations, which appeared to be line-dependent (Tsutsui and Higashiyama 2017). Unlike mutagenesis induced by CRISPR/Cas9, the outcome of Cre/*lox*-mediated recombination is unique, thus early events cannot be genetically distinguished from later events.

Further characterization of Cre/*lox*-mediated recombination in cowpea with *Cre* expressed by the *AtDD45*_*pro*_ verified that expressing *Cre* in the egg cells and young embryos was sufficient to complete excision in most if not all cells at a very early embryonic developmental stage. Compared with the *AtRps5a*_*pro*_, the *AtDD45*_*pro*_ has a more restricted expression profile limited to egg cells and young embryos before the eight-cell stage (Steffen et al. 2007; Lawit et al. 2013). Hence, Cre/*lox*-mediated recombination should end soon after the eight-cell stage if the *AtDD45*_*pro*_ maintained the same tissue-specificity in cowpea as in *Arabidopsis*. This would exclude the possibility of activating the reporter gene at later developmental stages. Considering that the genotyping results with p3785/p3786 indicated the absence of mosaic F_1_ progeny from the crosses with an *AtDD45*_*pro*_*Cre* line (R7743) pollinated by an *AtUbq3*_*pro*_*lox* line (3102), Cre/*lox*-mediated recombination should have completed in all cells at an early developmental stage despite expression of *Cre* over a shorter period. Even without mosaic F_1_ progeny identified in those crosses between line R7743 and line 3102, excision failure tended to occur more frequently than in the crosses between the *AtRps5a*_*pro*_*Cre* lines (1101, 1201, and 1203) and the same *AtUbq3*_*pro*_*lox* line (Table 3). Nevertheless, in the crosses between another *AtDD45*_*pro*_*Cre* line (R7715) and an *AtUbq10*_*pro*_*lox* line (R1719), excision occurred at a frequency as high as in the crosses between the *AtRps5a*_*pro*_*Cre* lines and the same *AtUbq10*_*pro*_*lox* line (Table 4), suggesting that differences in the recombination efficiency could be line-dependent and ascribed to other factors (e.g., integration and copy number) rather than the promoter activity *per se*. Differences in the pattern and efficiency of gene activation mediated by Cre/*lox* between lines have also been reported in tobacco (Odell et al. 1994) and maize (Zhang et al. 2003). The efficiency of gene activation with *Cre* expressed by the *AtDD45*_*pro*_ tended to vary more between lines and crosses compared with the *AtRps5a*_*pro*_ (Table 4), suggesting that an extended *Cre* expression could be important to complete gene activation for some lines with a lower recombination efficiency at the early developmental stage. Activation of the *lox*-reporter gene at later developmental stages by a prolonged expression of *Cre* in embryos and meristematic regions might make recombination failure at the early developmental stage undetectable in seedlings. As a result, the final recombination frequency in the *AtRps5a*_*pro*_*Cre* lines might have shown less variation than the *AtDD45*_*pro*_*Cre* lines in which such compensation would be absent. In addition, given that the coding sequence of *Cre* used in the *AtDD45*_*pro*_*Cre* cassette was codon-optimized, we cannot exclude the possibility that differences in the recombination efficiency between two constructs could also stem from differences between the coding sequences of the *Cre* genes which was not examined. Overall, shortening the expressing period of *Cre* did not necessarily decrease the efficiency of Cre/*lox*-mediated recombination, similar to the observation in *Arabidopsis* that the use of *AtRps5a*_*pro*_ and *AtDD45*_*pro*_ to express Cas9 ended in no significant difference in the efficiency of gene editing (Ordon et al. 2020).

The efficiency of Cre/*lox*-mediated recombination changed little in cowpea when the *Cre* lines served as a pollen donor. Among the crosses with the *lox*-reporter lines pollinated by the *Cre* lines, Cre/*lox*-mediated recombination mostly came about at a frequency comparable to their corresponding reciprocal crosses (Tables 3, 4), suggesting that the expression of *Cre* in the egg cells might not be essential for activating the reporter gene in every cell of F_1_ embryos as long as *Cre* was expressed after fertilization. Given that both *AtRps5a*_*pro*_ (Weijers et al. 2001; Maruyama et al. 2013) and *AtDD45*_*pro*_ (Steffen et al. 2007; Lawit et al. 2013) showed activity in zygotes and young embryos, expression of *Cre* during this post-fertilization period probably accounted for the gene activation in F_1_ embryos from crosses with the *Cre* lines as the paternal parent.

The original plan to use the *AtUbq3*_*pro*_ in the *lox*-reporter cassette to evaluate Cre/*lox*-mediated gene activation was unsuccessful because we could not observe *ZsGreen* expression in F_1_ seeds or seedlings. We thus designed and generated another *lox*-reporter cassette with the *AtUbq10*_*pro*_ expressing the reporter gene to evaluate gene activation in cowpea. The initial selection of the *AtUbq3*_*pro*_ was based on the strong *ZsGreen* expression in cowpea embryo axis tissue after microprojectile bombardment (Fig. S1c). Activation of the *ZsGreen* after co-bombardment with the cassettes *AtRps5a*_*pro*_*Cre* and *AtUbq3*_*pro*_*lox* also indicated that these two cassettes functioned in cowpea (Fig. S1d). However, *ZsGreen* activation was invisible in F_1_ mature seeds and seedlings but visible in some specific tissues (Figs. S3, S4) on F_1_ plants with the *lox*-flanked DNA sequence excised, suggesting either tissue-specificity for the *AtUbq3*_*pro*_ or some mechanisms of gene silencing in those lines that we did not explore. Although both the *AtUbq3*_*pro*_ and *AtUbq10*_*pro*_ were considered as “constitutive” promoters (Norris et al. 1993), expression from *AtUbq3* was shown to vary more than *AtUbq10* during plant development (Sun and Callis 1997; Schmid et al. 2005) and could even be inducible by dark (Sun and Callis 1997). The *AtUbq10* and *β-glucuronidase* controlled by the *AtUbq10*_*pro*_ were expressed much stronger than *AtUbq3* and *β-glucuronidase* controlled by the *AtUbq3*_*pro*_, respectively, across various tissue types analyzed (Schmid et al. 2005; Sun and Callis 1997), supporting our observation with the *lox*-reporter cassettes. Using the *AtUbq10*_*pro*_ to drive the reporter gene in the *lox*-reporter cassette made the outcome of Cre/*lox*-mediated recombination easy to screen in F_1_ seeds or seedlings. As a result, we could gauge the recombination efficiency by phenotyping F_1_ progeny based on *ZsGreen* expression rather than genotyping them to determine the excision of the *lox*-flanked intervening DNA sequence. The cassette *AtUbq10*_*pro*_*lox* contained a larger intervening DNA sequence between the *lox* sites than *AtUbq3*_*pro*_*lox*; however, the size of the intervening sequence between *lox* sites did not appear to affect recombination efficiency.

In summary, we characterized the Cre/*lox* gene activation system in both cowpea and tobacco and found that Cre/*lox*-mediated recombination could occur early and efficiently when *Cre* was controlled by *AtRps5a*_*pro*_ and *AtDD45*_*pro*_. The two-component gene activation system via Cre/*lox*-mediated recombination described here provides a useful tool for genetic engineering in cowpea and beyond.

## Supplementary Information

Below is the link to the electronic supplementary material.

Fig. S1 Expression of *ZsGreen* in cowpea embryo axes 24 h after microprojectile bombardment with two *AtUbq*_*3pro*_ expression cassettes; left column, PHP43648, *AtUbq*_*3pro*_:*ZsGreen:NOS*_*term*_; right column, pZZ006,* AtUbq*_*3pro*_*:lox:ZsGreen:NOS*_*term*_; a, b, bright field; c, d, FITC filter; bar = 1 mm. Supplementary file1 (TIFF 17886 KB)

Fig. S2 Expression of *AtUbq3* (AT5G03240) and *AtUbq10* (AT4G05320) in *Arabidopsis* across 47 tissue types obtained from the *Arabidopsis* eFP Browser (Schmid et al. 2005; Winter et al. 2007). Supplementary file2 (TIFF 24905 KB)

Fig. S3 Expression of *ZsGreen* in F_2_ seeds of cowpea from F_1_ plants derived from a cross between an *AtRps5a*_*pro*_*Cre* line (1101) and an *AtUbq3*_*pro*_*lox* line (3102). a, b, F_2_ from an F_1_ progeny with the lox-flanked PINIIterm excised; c, d, F_2_ from an F_1_ progeny without the *lox*-flanked* PINII*_*term*_ excised due to the absence of the *Cre*; a, c, bright field; b, d, FITC filter; bar = 5 mm. Supplementary file3 (TIFF 22527 KB)

Fig. S4 Expression of *ZsGreen* at the tip of peduncles from F_1_ plants of cowpea derived from a cross between an *AtRps5a*_*pro*_*Cre* line (1101) and an *AtUbq3*_*pro*_*lox* line (3102). a, b, F_1_ progeny with the *lox*-flanked * PINII*_*term*_ excised; c, d, F_1_ progeny without the lox-flanked * PINII*_*term*_ excised due to the absence of the* Cre*; a, c, bright field; b, d, FITC filter; bar = 5 mm. Supplementary file4 (TIFF 20094 KB)

Supplementary file5 (DOCX 37 KB)

## Data Availability

All data generated or analyzed during this study are included in this published article and its supplementary information files. Novel biological materials described in this publication may be available to the academic community and other not-for-profit institutions solely for non-commercial research purposes upon acceptance and signing of a material transfer agreement between the author's institution and the requestor. In some cases, such materials may originally contain genetic elements described in the manuscript that were obtained from a third party(s), and the authors may not be able to provide materials including third party genetic elements to the requestor because of certain third-party contractual restrictions placed on the author's institution. In such cases, the requester will be required to obtain such materials directly from the third party. The authors and authors' institution do not make any express or implied permission(s) to the requester to make, use, sell, offer for sale, or import third party proprietary materials. Obtaining any such permission(s) will be the sole responsibility of the requestor. Plant germplasm and transgenic material will not be made available except at the discretion of the owner and then only in accordance with all applicable governmental regulations.
