# Supplementary figures and images for: Gene activation via Cre/lox-mediated excision in cowpea (Vigna unguiculata)
Source: Plant Cell Rep. 2021 Sep 30;41(1):119–38. doi: 10.1007/s00299-021-02789-z (PMC8803690; doi:10.1007/s00299-021-02789-z)

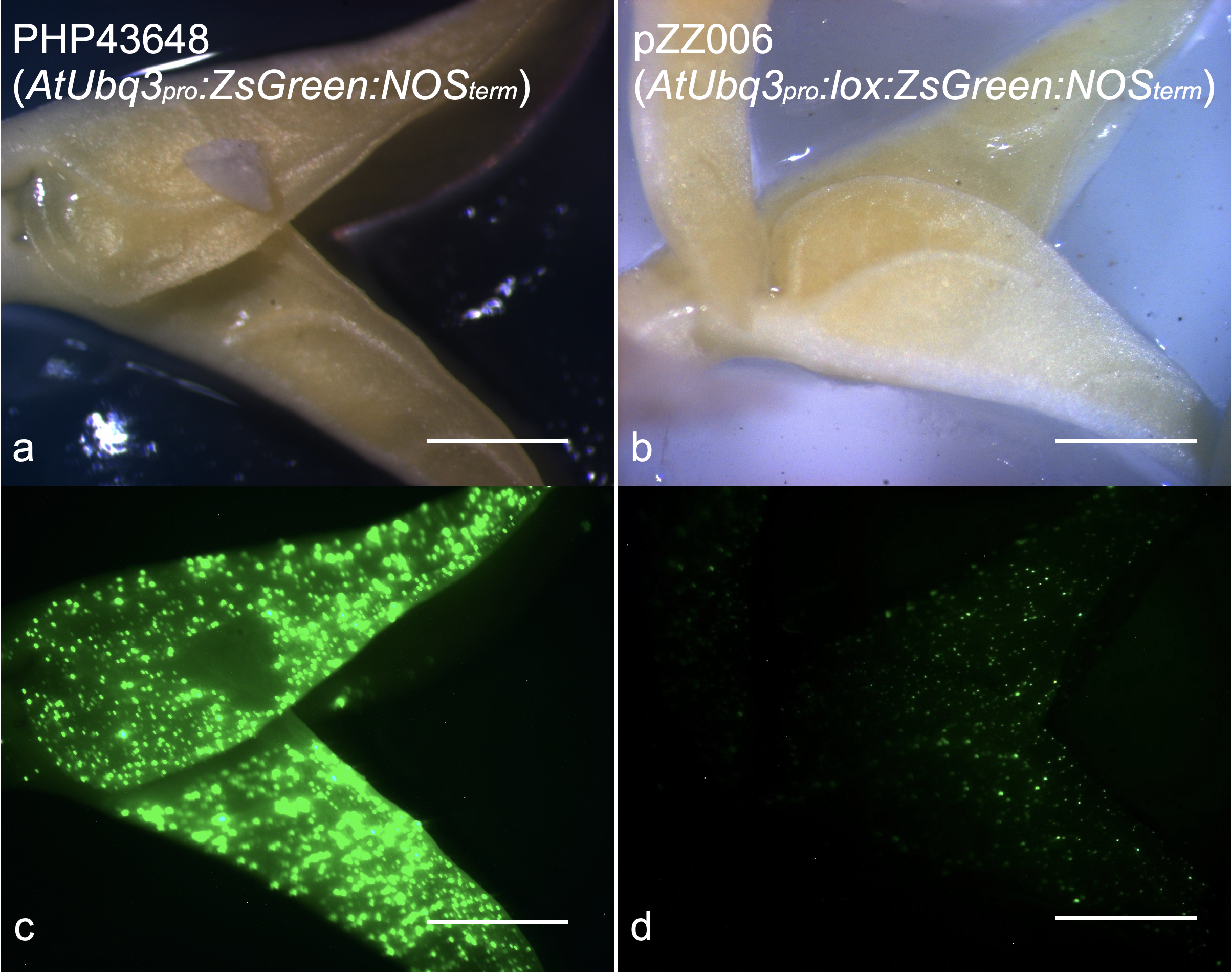

Supplement: Supplementary file 1 — Expression of ZsGreen in cowpea embryo axes 24 h after microprojectile bombardment with two AtUbq3pro expression cassettes; left column, PHP43648, AtUbq3pro:ZsGreen:NOSterm; right column, pZZ006, AtUbq3pro:lox:ZsGreen:NOSterm; a, b, bright field; c, d, FITC filter; bar = 1 mm. Supplementary file1 (TIFF 17886 KB) [file 299_2021_2789_MOESM1_ESM.tiff]

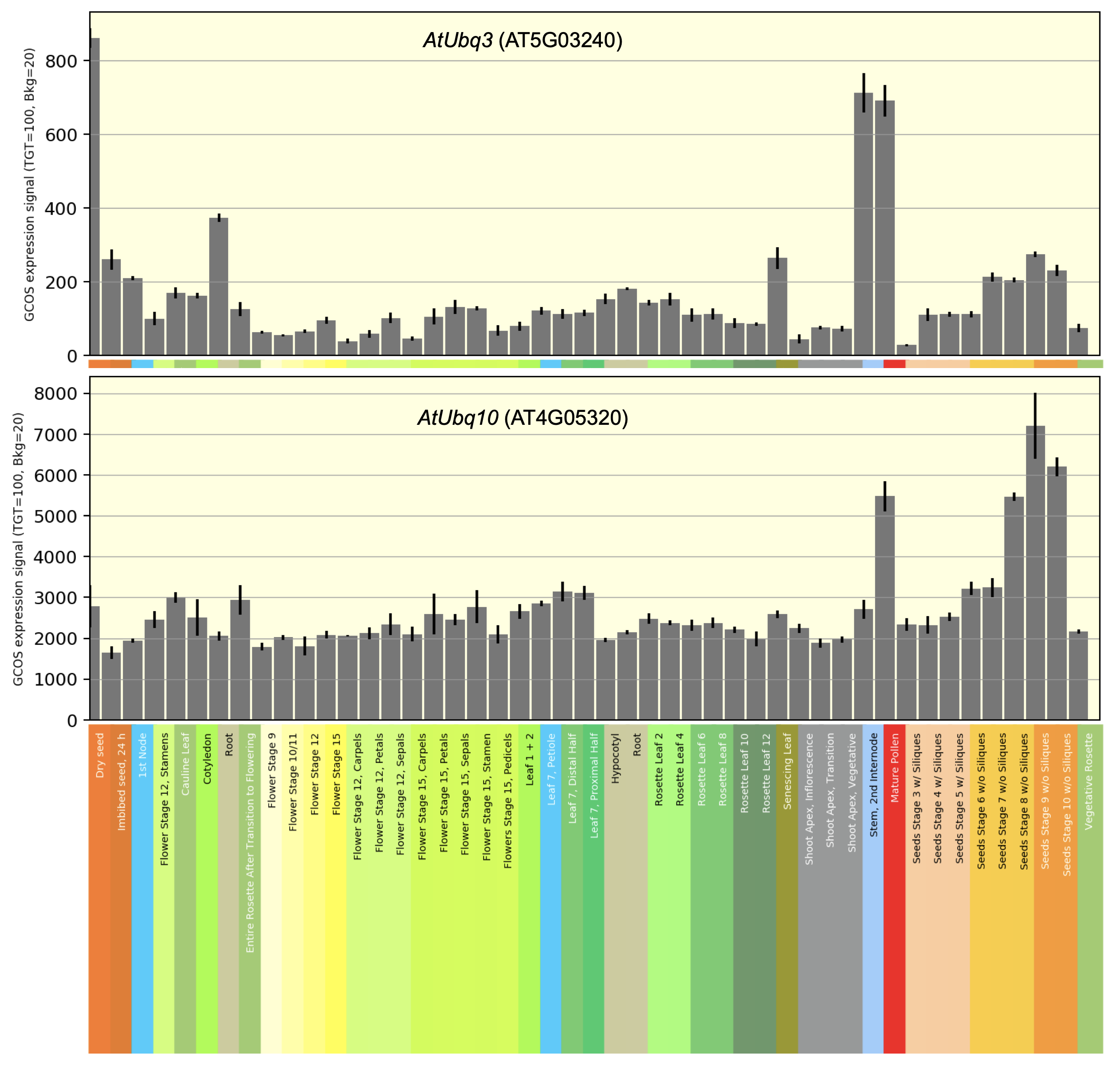

Supplement: Supplementary file 2 — Expression of AtUbq3 (AT5G03240) and AtUbq10 (AT4G05320) in Arabidopsis across 47 tissue types obtained from the Arabidopsis eFP Browser (Schmid et al. 2005; Winter et al. 2007). Supplementary file2 (TIFF 24905 KB) [file 299_2021_2789_MOESM2_ESM.tiff]

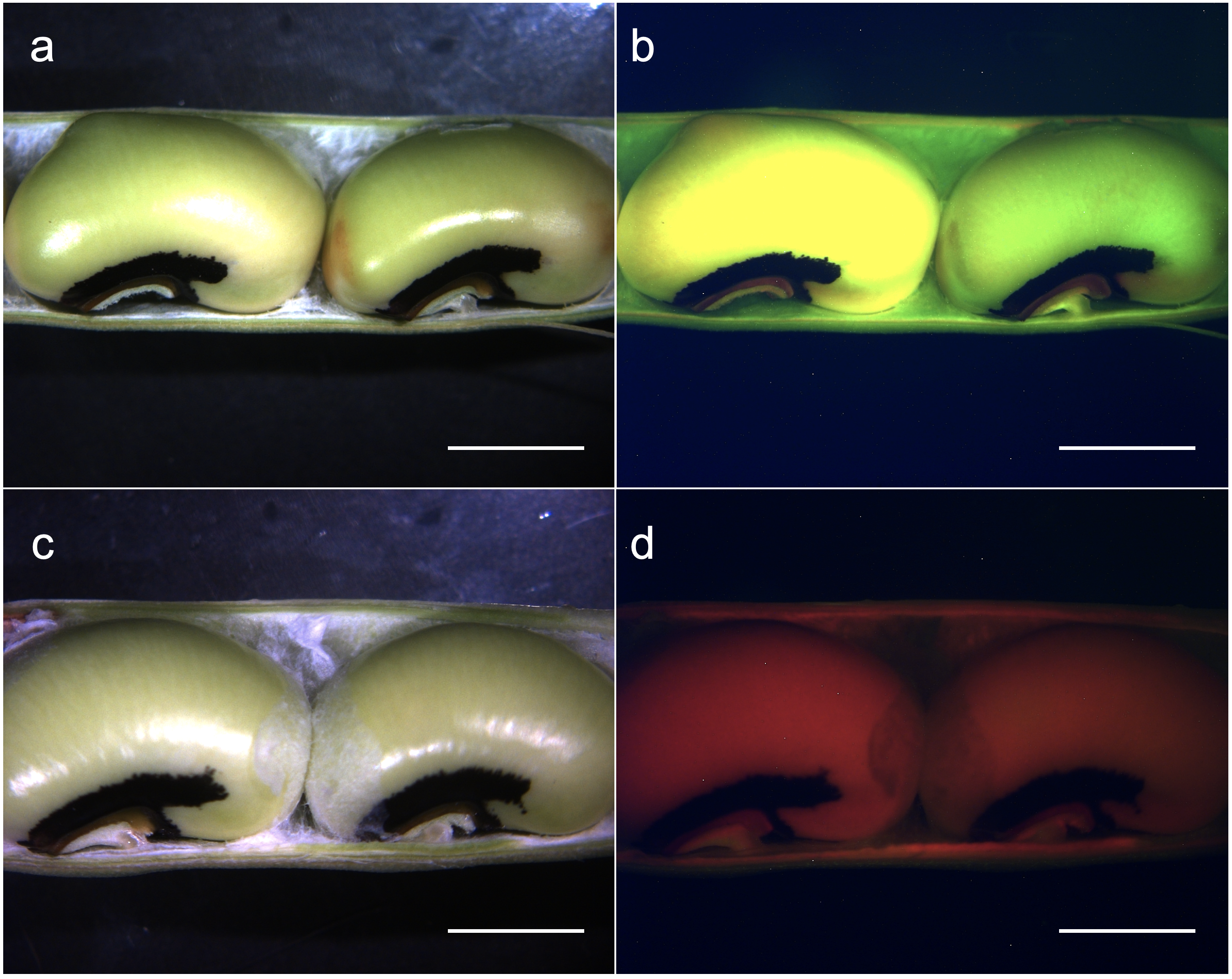

Supplement: Supplementary file 3 — Expression of ZsGreen in F2 seeds of cowpea from F1 plants derived from a cross between an AtRps5aproCre line (1101) and an AtUbq3prolox line (3102). a, b, F2 from an F1 progeny with the lox-flanked PINIIterm excised; c, d, F2 from an F1 progeny without the lox-flanked PINIIterm excised due to the absence of the Cre; a, c, bright field; b, d, FITC filter; bar = 5 mm. Supplementary file3 (TIFF 22527 KB) [file 299_2021_2789_MOESM3_ESM.tiff]

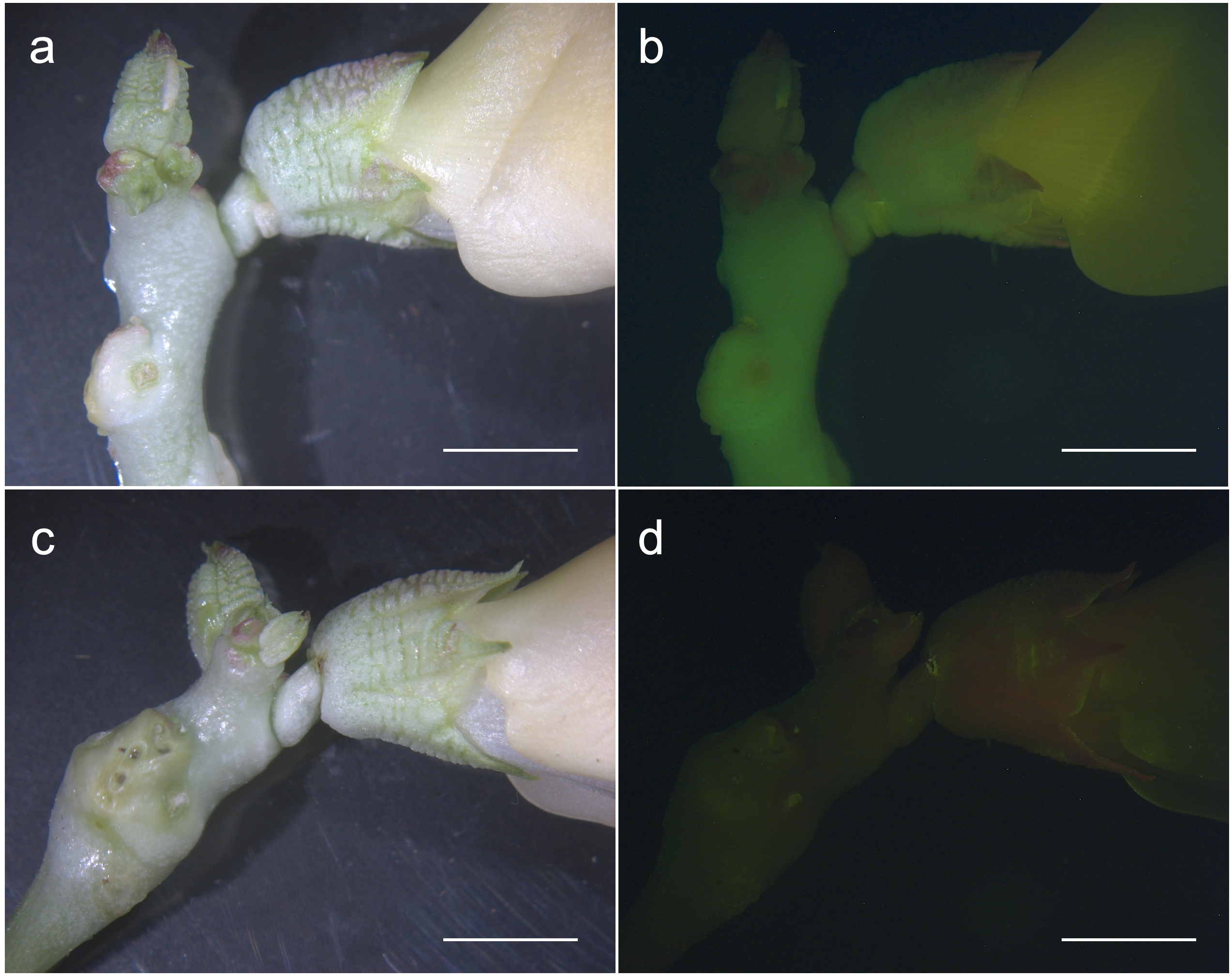

Supplement: Supplementary file 4 — Expression of ZsGreen at the tip of peduncles from F1 plants of cowpea derived from a cross between an AtRps5aproCre line (1101) and an AtUbq3prolox line (3102). a, b, F1 progeny with the lox-flanked PINIIterm excised; c, d, F1 progeny without the lox-flanked PINIIterm excised due to the absence of the Cre; a, c, bright field; b, d, FITC filter; bar = 5 mm. Supplementary file4 (TIFF 20094 KB) [file 299_2021_2789_MOESM4_ESM.tiff]
