# Supplementary material for: Gene activation via Cre/lox-mediated excision in cowpea (Vigna unguiculata)
Source: Plant Cell Rep. 2021 Sep 30;41(1):119–38. doi: 10.1007/s00299-021-02789-z (PMC8803690; doi:10.1007/s00299-021-02789-z)
Supplement: Supplementary file 5 — Supplementary file5 (DOCX 37 KB) [file 299_2021_2789_MOESM5_ESM.docx]

Table S1 Vectors used for plant transformation.

| Vector ID | Binary vector backbone | Cassettes in T-DNA^a^ |
| --- | --- | --- |
| pZZ010 | pPZP201BK | *LB- StUbq3_pro_:hpt:NOS_term_ // AtRps5a_pro_:Cre:phaseolin_term_ // GmEF1a_pro_:DsRED:NOS_term_ -RB* |
| pZZ011 | pPZP201BK | *LB- StUbq3_pro_:bar:NOS_term_ // AtRps5a_pro_:Cre:phaseolin_term_ // GmEF1a_pro_:DsRED:NOS_term_ -RB* |
| pZZ012 | pPZP201BK | *LB- GmEF1a_pro_:DsRED:NOS_term_ // StUbq3_pro_:nptII:NOS_term_ // AtRps5a:Cre:phaseolin_term_ -RB* |
| pZZ017 | pORE | *LB- AtUbq3_pro_:lox-PINII_term_-lox:ZsGreen:NOS_term_ // S1_pro_:nptII:S3_term_ -RB* |
| pZZ031B | pPZP201BK | *LB- GmEF1a_pro_:DsRED:NOS_term_ // S1_pro_:nptII:S3_term_ // AtUbq3_pro_:lox-PINII_term_-lox:ZsGreen:NOS_term_ -RB* |
| RC2677 | pAGM4673 | *LB- 2×CaMV35S_enhancer_:CaMV35S_pro_:TMV𝜔_translation enhancer_:CTP:aadA1:CaMV35S_term_ // TBS insulator // AtDD45_pro_:Cre:OCS_term_ -RB* |
| RC2717 | pAGM4673 | *LB- 2×CaMV35S_enhancer_:CaMV35S_pro_:TMV𝜔_translation enhancer_:CTP:aadA1:CaMV35S_term_, //  AtUbq10_pro_:loxP-GmUbq3_pro_:ER-SP:tdTomatoER:KDEL:OCS_term_ -loxP:ER-SP:ZsGreen: KDEL:NOS -RB* |

^a^CTP, Chloroplast transit peptide; ER-SP, ER signal peptide; KDEL, KDEL-ER retention motif; LB, left border; RB, right border.

Table S2 Media used in cowpea transformation by Method 1 and Method 2.

|  | Co-culture medium (CCM) | Modified co-culture medium (CCM’) | Shoot induction medium (SIM) | Shoot elongation medium (SEM) | Germination medium (GM) |
| --- | --- | --- | --- | --- | --- |
| MS salts | 1× | 1× | 1× | 1× | 1× |
| MS vitamins | 1/10× | 1/10× | 1× | 1× | 1× |
| MES | 20 mM | 20 mM | 3 mM | 3 mM | 3 mM |
| Sucrose (w/v) | 3% | 3% | 3% | 3% | 3% |
| Agar^a^ | 8 g/l | 8 g/l | 8 g/l | 8 g/l | 8 g/l |
| pH | 5.4 | 5.4 | 5.6 | 5.6 | 5.6 |
| 6-benzylaminopurine (BA) | 1.67 mg/l | 1.67 mg/l | 1.67 mg/l |  | 5 mg/l |
| Gibberellic acid (GA3) | 0.25 mg/l | 0.25 mg/l |  | 0.5 mg/l |  |
| Indole-3-acetic acid (IAA) |  |  |  | 0.1 mg/l |  |
| Sodium thiosulphate | 1 mM | 1 mM |  |  |  |
| Dithiothreitol (DTT) | 1 mM | 1 mM |  |  |  |
| L-cysteine |  | 400 mg/l |  |  |  |
| Acetosyringone (AS) | 0.2 mM | 0.2 mM |  |  |  |
| asparagine |  |  |  | 50 mg/l |  |
| meropenem |  |  | 30 mg/l | 30 mg/l |  |

^a^Agar was omitted from liquid media

Table S3 Primers used for genotyping transgenic events and F_1_ progeny.

| Primer ID | Primer sequence 5’ to 3’ | Targeted sequence | Size (bp) | Annealing temp (°C) |
| --- | --- | --- | --- | --- |
| p3753 | CACACTGCAGATGTCCAATTTACTGACCGTACAC | *Cre:phaseolin_term_* in the cassette *AtRps5a_pro_Cre* | 2221 | 60 |
| p3768 | GAGAAAGCTTGGCGCGCCTGAAACATCCCTGAAGTGTCTCA |  |  |  |
| p3769 | CAGCGTTTTCGTTCTGCCAA | *Cre* in the cassette *AtRps5a_pro_Cre*^a^ | 184 | 60 |
| p3770 | CGACCAGGTTCGTTCACTCA |  |  |  |
| p3753 | CACACTGCAGATGTCCAATTTACTGACCGTACAC | *Cre* in the cassette *AtRps5a_pro_Cre* | 619 | 60 |
| p3769 | CAGCGTTTTCGTTCTGCCAA |  |  |  |
| p3785 | TTTCTTTGTTCGATTCTCTCTGT | *AtUbq3_intron1_* (151 bp)*::lox-PINII_term_-lox::ZsGreen::NOS_term_* in the cassette *AtUbq3_pro_lox* | 1562 | 57 |
| p3908 | GAGAGGCGCGCCCGATCTAGTAACATAGATGACACCG |  |  |  |
| p3785 | TTTCTTTGTTCGATTCTCTCTGT | *AtUbq3_intron1_* (151 bp)*::lox-PINII_term_-lox::ZsGreen* (146 bp) in the cassette *AtUbq3_pro_lox* | 699 | 57 |
| p3786 | ACCACGCACAGGTTGATGG |  |  |  |
| p3700 | TGTACACGGTGTCGAACTGG | *ZsGreen* in the cassette *AtUbq3_pro_lox*^a^ | 189 | 60 |
| p3701 | AGTCCAAGTTCTACGGCGTG |  |  |  |
| p4257 | GGCCATCAGCTGGGAAGTT | *ZsGreen* (codon-optimized) in the cassette *AtUbq10_pro_lox* | 399 | 60 |
| p4258 | AGTCCAAGCACGGCCTTAC |  |  |  |
| p4261 | GCCTGGCTGTCAAGACCAT | *Cre* (codon-optimized) in the cassette *AtDD45_pro_Cre* | 705 | 60 |
| p4262 | CTGTGATGTGGCTGACGGT |  |  |  |
| p4793 | AAATGTTCCTCGCTGACGTA | Full-length of the cassette *AtDD45_pro_Cre* | 2937 | 60 |
| p4795 | CTGCTGAGCCTCGACATGT |  |  |  |
| p4794 | ATGATGAGCAATCTATTGACCGT | *Cre:OCS_term_* in the cassette *AtDD45_pro_Cre* | 1934 | 60 |
| p4795 | CTGCTGAGCCTCGACATGT |  |  |  |
| p4796 | ATGGCTCAGTCCAAGCACG | *ZsGreen:NOS_term_* in the cassette *AtUbq10_pro_lox* | 973 | 60 |
| p4797 | GATCTAGTAACATAGATGACACCGC |  |  |  |
| p4797 | GATCTAGTAACATAGATGACACCGC | Full length of the cassette *AtUbq10_pro_lox* | 5822 | 60 |
| p4798 | GACGTCGTTGTGGTTGGTG |  |  |  |
| p4825 | CAATTAGCCAAAAACAACTTTGCGT | *AtUbq10_pro(141bp)+intron1_::lox- GmUbq3_pro+intron1_::tdTomatoER:OCS_term_ -lox::ZsGreen*(403 bp) in the cassette *AtUbq10_pro_lox* | 4282 | 60 |
| p4826 | CATCAGCTGGGAAGTTCACG |  |  |  |

^a^primer sets were used to generate DIG-labeled probe.

Table S4 Primers used in quantitative PCR.

| Primer ID | Primer sequence 5’ to 3’ | Gene | Size (Efficiency) |
| --- | --- | --- | --- |
| p3766 | AAGCCGGTCTTGTCGATCAG | *nptII* | 165 bp |
| p3767 | AAAAGCGGCCATTTTCCACC |  | (1.96) |
| p4133 | GTACATGGCCTGTTGTTTGATG | *NtTubulin*, predicted tobacco tubulin α chain-like (XM_016623993.1) | 94 bp |
| p4134 | CTGGATGGTCCTCTTTGTCTTT |  | (1.97) |
| p4225 | GGTTGATTCCTTGAAGAGAAGC | *VuFbox*, predicted cowpea F-Box protein (Vigun07g146600) | 130 bp |
| p4226 | TGCTTGCCTATCCCAGTTAAG |  | (2.02) |
| p4229 | TGACGGTGGGAGAATGTTAATC | *Cre*, in the cassette *AtRps5a_pro_Cre* | 129 bp |
| p4230 | GCTACACCAGAGACGGAAATC |  | (1.96) |
| p4231 | GACGGCTCCTTCATCTACAAG | *DsRED*, in the cassette *GmEF1a_pro_DsRED* | 147 bp |
| p4232 | CTTGTGGATCTCGCCCTTC |  | (1.90) |
| p4233 | CTACTTCAAGAACTCCTGCCC | *ZsGreen*, in the cassette *AtUbq3_pro_loxP* | 126 bp |
| p4234 | TCGTGGTACATGCAGTTCTC |  | (1.95) |
| p4259 | GGCCATCAGCTGGGAAGTT | *ZsGreen* (codon-optimized) in the cassette *AtUbq10_pro_loxP* | 114 bp |
| p4260 | TTTTCGAGGATGGCGCTGT |  | (1.96) |
| p4263 | CTGGAAGATGCTTTTGAGCG | *Cre* (codon-optimized), in the cassette *AtDD45_pro_Cre* | 144 bp |
| p4264 | TGAATGGTCTTGACAGCCAG |  | (2.00) |

Note: the annealing temperature of all primer combinations is 60°C.

Table S5 Transgene segregation in tobacco T1 progeny from *AtRps5a_pro_Cre* lines and the *AtUbq3_pro_lox* line.

| Line ID | Cassette | # transgenic seedlings | # non-transgenic seedlings | Segregation ratio (p-value^a^) |
| --- | --- | --- | --- | --- |
| pZZ010_1.1 | *AtRps5a_pro_Cre* | 169 | 45 | 3:1 (0.18) |
| pZZ010_1.3 | *AtRps5a_pro_Cre* | 242 | 0 | 255:1 (0.33) |
| pZZ010_2.2 | *AtRps5a_pro_Cre* | 212 | 14 | 15:1 (0.97) |
| pZZ010_202.3 | *AtRps5a_pro_Cre* | 158 | 50 | 3:1 (0.75) |
| pZZ010_201.2 | *AtRps5a_pro_Cre* | 206 | 7 | 15:1 (0.07) |
| pZZ012_1.4 | *AtRps5a_pro_Cre* | 210 | 13 | 15:1 (0.80) |
| pZZ012_2.1 | *AtRps5a_pro_Cre* | 234 | 14 | 15:1 (0.69) |
| pZZ017_1.1 | *AtUbq3_pro_loxP* | 562 | 1 | 255:1 (0.42) |

^a^Segregation ratios were tested by Chi-square.

Table S6 Identification of homozygous tobacco T1 progeny from two single-locus *AtRps5a_pro_Cre* lines.

| T1 Plant ID | Line ID | # transgenic T2 seedlings (Hyg+ & DsRed+) | # non-transgenic T2 seedlings (Hyg− & DsRed−) | Zygosity  (segregation ratio^a^) |
| --- | --- | --- | --- | --- |
| pZZ010_1.1_1 | pZZ010_1.1 | 226 | 0 | homozygote |
| pZZ010_1.1_2 | pZZ010_1.1 | 109 | 32 | hemizygote (3:1, 0.53) |
| pZZ010_1.1_3 | pZZ010_1.1 | 313 | 0 | homozygote |
| pZZ010_1.1_4 | pZZ010_1.1 | 430 | 0 | homozygote |
| pZZ010_1.1_5 | pZZ010_1.1 | 125 | 30 | hemizygote (3:1, 0.10) |
| pZZ010_1.1_7 | pZZ010_1.1 | 165 | 0 | homozygote |
| pZZ010_1.1_9 | pZZ010_1.1 | 128 | 32 | hemizygote (3:1, 0.14) |
| pZZ010_1.1_10 | pZZ010_1.1 | 233 | 0 | homozygote |
|  |  |  |  |  |
| pZZ010_202.3_11 | pZZ010_202.3 | 48 | 0 | homozygote |
| pZZ010_202.3_13 | pZZ010_202.3 | 142 | 39 | hemizygote (3:1, 0.28) |
| pZZ010_202.3_14 | pZZ010_202.3 | 110 | 28 | hemizygote (3:1, 0.20) |
| pZZ010_202.3_15 | pZZ010_202.3 | 174 | 49 | hemizygote (3:1, 0.30) |

^a^Segregation ratios were tested by Chi-square.

Table S7 Cre/*lox*-mediated gene activation in tobacco F_1_ embryos from crosses with homozygous T1 *AtRps5a_pro_Cre* lines pollinated by T1 *AtUbq3_pro_loxP* lines.

| Female parent | Male parent | # ZsGreen+ embryos | # ZsGreen− embryos | # total embryos observed | % ZsGreen+ embryos | Embryo developmental stage |
| --- | --- | --- | --- | --- | --- | --- |
| pZZ010_1.1_1 | pZZ017_1.1_4.2 | 14 | 91 | 105 | 13.3 | Globular, pre-globular |
| pZZ010_1.1_1 | pZZ017_1.1_7.1 | 11 | 54 | 65 | 16.9 | Globular, pre-globular |
| pZZ010_1.1_3 | pZZ017_1.1_7.1 | 7 | 92 | 99 | 7.1 | Globular, heart |
| pZZ010_1.1_4 | pZZ017_1.1_4.2 | 44 | 67 | 111 | 39.6 | Globular, heart |
| pZZ010_1.1_4 | pZZ017_1.1_7.1 | 34 | 77 | 111 | 30.6 | Globular, heart |
| pZZ010_1.1_4 | pZZ017_1.1_8.1 | 35 | 15 | 50 | 70.0 | Globular, pre-globular |

Table S8 Estimate of transgene copy number in cowpea T0 transgenic lines by qPCR.

| Line ID | Cassette (vector) | # T0 plants surviving | Ratio of target gene to reference gene (*VuFbox*)^a^ | copy # of transgene estimated by qPCR |
| --- | --- | --- | --- | --- |
| 1201 | *AtRps5a_pro_Cre* (pZZ012) | 8 | 0.4 ± 0.02 | 1 |
| 1202 | *AtRps5a_pro_Cre* (pZZ012) | 5 | 2.0 ± 0.05 | 4 |
| 1203 | *AtRps5a_pro_Cre* (pZZ012) | 9 | 3.7 ± 0.12 | 6-8 |
| 1101 | *AtRps5a_pro_Cre* (pZZ011) | 3 | 0.7 ± 0.06 | 1-2 |
| 3101 | *AtUbq3_pro_lox* (pZZ031B) | 10 | 3.0 ± 0.12 | 5-6 |
| 3102 | *AtUbq3_pro_lox* (pZZ031B) | 1 | 0.5 | 1 |
| R770101 | *AtDD45_pro_Cre* (RC2677) | 1 | 3.6 | 7 |
| R770102 | *AtDD45_pro_Cre* (RC2677) | 1 | 1.6 | 3 |
| R7702 | *AtDD45_pro_Cre* (RC2677) | 1 | 4.8 | 9-10 |
| R7714 | *AtDD45_pro_Cre* (RC2677) | 2 | 1.7 ± 0.17 | 3-4 |
| R7715 | *AtDD45_pro_Cre* (RC2677) | 1 | 1.2 | 2-3 |
| R7719 | *AtDD45_pro_Cre* (RC2677) | 1 | 0.4 | 1 |
| R7743 | *AtDD45_pro_Cre* (RC2677) | 1 | 0.7 | 1-2 |
| R7744 | *AtDD45_pro_Cre* (RC2677) | 1 | 1.8 | 3-4 |
| R7747 | *AtDD45_pro_Cre* (RC2677) | 1 | 2.1 | 4 |
| R170102 | *AtUbq10_pro_lox* (RC2717) | 1 | 0.3 | 1 |
| R170103 | *AtUbq10_pro_lox* (RC2717) | 1 | 1.0 | 2 |
| R1702 | *AtUbq10_pro_lox* (RC2717) | 1 | 0.6 | 1-2 |
| R1704 | *AtUbq10_pro_lox* (RC2717) | 1 | 1.3 | 2-3 |
| R1713 | *AtUbq10_pro_lox* (RC2717) | 1 | 0.5 | 1 |
| R1714 | *AtUbq10_pro_lox* (RC2717) | 1 | 0.9 | 1-2 |
| R1716 | *AtUbq10_pro_lox* (RC2717) | 1 | 0.5 | 1 |
| R1719 | *AtUbq10_pro_lox* (RC2717) | 1 | 1.2 | 2-3 |
| R1720 | *AtUbq10_pro_lox* (RC2717) | 1 | 0.8 | 1-2 |
| R1723 | *AtUbq10_pro_lox* (RC2717) | 1 | 1.6 | 3-4 |
| R1725 | *AtUbq10_pro_lox* (RC2717) | 1 | 3.1 | 6 |
| R1739 | *AtUbq10_pro_lox* (RC2717) | 3 | 0.7 ± 0.04 | 1-2 |
| R1744 | *AtUbq10_pro_lox* (RC2717) | 1 | 1.2 | 2-3 |

^a^Data for the lines with multiple T0 plants surviving are means ± standard error.

Table S9 Transgene segregation in cowpea T1 progeny of two single-locus *AtRps5a_pro_Cre* lines.

| Plant ID | Line ID | Vector | # DsRed+ seeds | # DsRed− seeds | # total seeds | Segregation ratio  (p-value^a^) | Copy # estimated based on qPCR (*Cre/VuFbox*) |
| --- | --- | --- | --- | --- | --- | --- | --- |
| 110104 | 1101 | pZZ011 | 194 | 56 | 250 | 3:1 (0.342) | 0.9 |
| 120107 | 1201 | pZZ012 | 335 | 95 | 430 | 3:1 (0.16) | 0.5 |

^a^Segregation ratios were tested by Chi-square.

Table S10 Transgene segregation in cowpea T1 progeny of *AtDD45_pro_Cre* lines and *AtUbq10_pro_lox* lines.

| T0 Plant ID | Line ID | Vector | # transgenic T1 | # non-transgenic T1 | # total T1 screened | Segregation ratio  (p-value^a^) | Copy # estimated based on qPCR (target gene^b^/*VuFbox*) | |
| --- | --- | --- | --- | --- | --- | --- | --- | --- |
| R771402 | R7714 | RC2677 | 17 | 7 | 24 | 3:1 (0.64) | 2-3 (1.5) |  |
| R771501 | R7715 | RC2677 | 29 | 7 | 36 | 3:1 (0.44) | 2-3 (1.2) |  |
| R774301 | R7743 | RC2677 | 53 | 18 | 71 | 3:1 (0.95) | 1-2 (0.7) |  |
| R170102 | R170102 | RC2717 | 31 | 27 | 58 | 1:1 (0.60) | 1 (0.3) |  |
| R170103 | R170103 | RC2717 | 32 | 3 | 35 | 15:1 (0.57) | 2 (1.0) |  |
| R170202 | R1702 | RC2717 | 133 | 31 | 164 | 3:1 (0.07) | 1-2 (0.6) |  |
| R171301 | R1713 | RC2717 | 16 | 36 | 52 | 1:1 (0.006) | 1 (0.5) |  |
| R171401 | R1714 | RC2717 | 38 | 5 | 43 | 15:1 (0.15) | 1-2 (0.9) |  |
| R171901 | R1719 | RC2717 | 68 | 32 | 100 | 3:1 (0.11) | 2-3 (1.2) |  |
| R172001 | R1720 | RC2717 | 25 | 20 | 45 | 1:1 (0.46) | 1-2 (0.8) |  |
| R173901 | R1739 | RC2717 | 87 | 19 | 106 | 3:1 (0.09) | 1-2 (0.7) |  |
| R173902 | R1739 | RC2717 | 43 | 15 | 57 | 3:1 (0.88) | 1-2 (0.7) |  |
| R173903 | R1739 | RC2717 | 50 | 14 | 64 | 3:1 (0.56) | 1-2 (0.8) |  |

^a^Segregation ratios were tested by Chi-square.

^b^The target gene was *Cre* for vector RC2677 and was *ZsGreen* for vector RC2717.

Table S11 Identification of homozygous cowpea T1 progeny from two single-locus *AtRps5a_pro_Cre* lines.

| T1 Plant ID | Line ID | # DsRed+ T2 seeds | # DsRed- T2 seeds | # total seeds | Zygosity | Segregation ratio (p-value^a^) |
| --- | --- | --- | --- | --- | --- | --- |
| 110104_1 | 1101 | 221 | 147 | 368 | hemizygote | 3:1 (<0.001) |
| 110104_2 | 1101 | 171 | 0 | 171 | homozygote |  |
| 110104_3 | 1101 | 299 | 0 | 299 | homozygote |  |
| 110104_4 | 1101 | 187 | 0 | 187 | homozygote |  |
| 110104_5 | 1101 | 174 | 80 | 254 | hemizygote | 3:1 (<0.001) |
| 110104_6 | 1101 | 166 | 0 | 166 | homozygote |  |
| 120105_1 | 1201 | 359 | 0 | 359 | homozygote |  |
| 120105_2 | 1201 | 169 | 94 | 263 | hemizygote | 3:1 (<0.001) |
| 120105_3 | 1201 | 202 | 71 | 273 | hemizygote | 3:1 (0.70) |
| 120105_4 | 1201 | 220 | 0 | 220 | homozygote |  |
| 120105_5 | 1201 | 40 | 25 | 65 | hemizygote | 3:1 (0.01) |
| 120105_6 | 1201 | 29 | 0 | 29 | homozygote |  |
| 120106_1 | 1201 | 145 | 59 | 204 | hemizygote | 3:1 (0.20) |
| 120107_1 | 1201 | 283 | 0 | 283 | homozygote |  |
| 120107_2 | 1201 | 348 | 130 | 478 | hemizygote | 3:1 (0.27) |
| 120107_3 | 1201 | 167 | 55 | 222 | hemizygote | 3:1 (0.94) |
| 120107_4 | 1201 | 123 | 57 | 180 | hemizygote | 3:1 (0.04) |
| 120107_5 | 1201 | 92 | 26 | 118 | hemizygote | 3:1 (0.46) |
| 120107_6 | 1201 | 255 | 0 | 255 | homozygote |  |

^a^Segregation ratios were tested by Chi-square.

Table S12 Identification of homozygous cowpea T1 progeny from a single-locus *AtUbq3_pro_lox* line by qPCR.

| Plant ID | Line ID | Ratio of *ZsGreen/VuFbox* based on qPCR | Zygosity |
| --- | --- | --- | --- |
| 310200 T0 |  | 0.5 | hemizygote |
| 310200_1 | 3102 | 0.6 | hemizygote |
| 310200_2 | 3102 | 0.5 | hemizygote |
| 310200_3 | 3102 | 0.6 | hemizygote |
| 310200_4 | 3102 | 0.6 | hemizygote |
| 310200_6 | 3102 | 0.5 | hemizygote |
| 310200_7 | 3102 | 0.7 | hemizygote |
| 310200_8 | 3102 | 1.4 | **homozygote** |
| 310200_9 | 3102 | 1.4 | **homozygote** |
| 310200_10 | 3102 | 0.8 | hemizygote |
| 310200_11 | 3102 | 0.8 | hemizygote |
| 310200_12 | 3102 | 0.7 | hemizygote |

Table S13 Homozygous cowpea T1 progeny identified from *AtDD45_pro_Cre* lines and *AtUbq10_pro_lox* lines by qPCR.

| Plant ID | Line ID | Vector | Ratio of target gene^a^*/VuFbox* based on qPCR | Zygosity |
| --- | --- | --- | --- | --- |
| R774301 T0 | R7743 | RC2677 | 0.6 | hemizygote |
| R774301_55 | R7743 | RC2677 | 1.3 | homozygote |
| R774301_57 | R7743 | RC2677 | 1.3 | homozygote |
| R774301_61 | R7743 | RC2677 | 1.9 | homozygote |
| R774301_63 | R7743 | RC2677 | 1.2 | homozygote |
| R774301_65 | R7743 | RC2677 | 1.9 | homozygote |
| R774301_69 | R7743 | RC2677 | 1.9 | homozygote |
| R774301_83 | R7743 | RC2677 | 1.6 | homozygote |
| R771501 T0 | R7715 | RC2677 | 0.6 | hemizygote |
| R771501_23 | R7715 | RC2677 | 1.0 | homozygote |
| R771402 T0 | R7714 | RC2677 | 0.7 | hemizygote |
| R771402_21 | R7714 | RC2677 | 1.8 | homozygote |
| R771402_24 | R7714 | RC2677 | 1.7 | homozygote |
| R170202 T0 | R1702 | RC2717 | 0.7 | hemizygote |
| R170202_5 | R1702 | RC2717 | 1.4 | homozygote |
| R170202_7 | R1702 | RC2717 | 1.9 | homozygote |
| R173902 T0 | R1739 | RC2717 | 0.7 | hemizygote |
| R173902_1 | R1739 | RC2717 | 1.3 | homozygote |
| R173902_6 | R1739 | RC2717 | 1.4 | homozygote |
| R173902_7 | R1739 | RC2717 | 1.4 | homozygote |
| R173902_8 | R1739 | RC2717 | 1.5 | homozygote |
| R173902_13 | R1739 | RC2717 | 1.5 | homozygote |
| R173902_14 | R1739 | RC2717 | 1.5 | homozygote |
| R171901 T0 | R1719 | RC2717 | 0.6 | hemizygote |
| R171901_7 | R1719 | RC2717 | 1.2 | homozygote |
| R171901_8 | R1719 | RC2717 | 1.3 | homozygote |
| R171901_11 | R1719 | RC2717 | 1.2 | homozygote |
| R171901_12 | R1719 | RC2717 | 1.3 | homozygote |
| R171901_13 | R1719 | RC2717 | 1.3 | homozygote |
| R171901_16 | R1719 | RC2717 | 1.3 | homozygote |

^a^The target gene was *Cre* for vector RC2677 and *ZsGreen* for vector RC2717.

Table S14 Identification of homozygous cowpea T1 progeny from two multi-copy *AtRps5a_pro_Cre* lines.

| T1 Plant ID | Line ID | Copy # of transgene based on qPCR (*Cre/VuFbox*) | # DsRed+ T2 seedlings | # DsRed- T2 seedlings | # total seedlings germinated | Zygosity | Segregation ratio  (p-value^a^) |
| --- | --- | --- | --- | --- | --- | --- | --- |
| 120200_1 | 1202 | 2 (1.1) | 26 | 20 | 46 | hemizygote | 3:1 (<0.001) |
| 120200_9 | 1202 | 2 (1.0) | 32 | 12 | 44 | hemizygote | 3:1 (0.73) |
| 120200_11 | 1202 | 2 (1.0) | 15 | 16 | 31 | hemizygote | 3:1 (<0.001) |
| 120200_13 | 1202 | 2 (0.9) | 29 | 18 | 47 | hemizygote | 3:1 (0.04) |
| 120200_14 | 1202 | 2 (0.9) | 34 | 16 | 50 | hemizygote | 3:1 (0.25) |
| 120200_16 | 1202 | 2 (0.9) | 36 | 17 | 53 | hemizygote | 3:1 (0.23) |
| 120200_18 | 1202 | 2 (0.9) | 34 | 15 | 49 | hemizygote | 3:1 (0.36) |
| 120200_20 | 1202 | 2 (0.9) | 38 | 11 | 49 | hemizygote | 3:1 (0.68) |
| 120300_4 | 1203 | 1-2 (0.7) | 35 | 16 | 51 | hemizygote | 3:1 (0.29) |
| 120300_5 | 1203 | 1-2 (0.8) | 32 | 10 | 42 | hemizygote | 3:1 (0.86) |
| 120300_6 | 1203 | 1-2 (0.8) | 34 | 10 | 44 | hemizygote | 3:1 (0.73) |
| 120300_7 | 1203 | 1-2 (0.7) | 50 | 0 | 50 | **homozygote** |  |
| 120300_8 | 1203 | 1 (0.45) | 31 | 15 | 46 | hemizygote | 3:1 (0.23) |

^a^Segregation ratios were tested by Chi-square.
